# Sparingly PEGylated Adipate Copolymers via Enzymatic Synthesis as Nano-Carriers for Solid Dispersions

**DOI:** 10.1021/acs.biomac.6c00647

**Published:** 2026-06-05

**Authors:** Eleni Axioti, Nana A. Berfi, Philippa L. Jacob, Klara M. Saller, Georgia L. Maitland, Anisha Patel, Sri Nithya Paruchuri, Paul D. Topham, Matthew J. Derry, Shreyasi Chatterjee, Benoit Couturaud, Luciano Galantini, Iolanda Francolini, Valentina Cuzzucoli Crucitti, Veeren M. Chauhan, Robert J. Cavanagh, Vincenzo Taresco

**Affiliations:** † School of Chemistry, University Park, Nottingham NG7 2RD, U.K.; ‡ School of Pharmacy, 6123 University of Nottingham, Boots Sciences Building, University Park, Nottingham NG7 2RD, U.K.; § Institute for Chemical Technology of Organic Materials, 27266 Johannes Kepler University Linz, Altenbergerstrasse, 69 4040 Linz, Austria; ∥ Department of Chemical Engineering and Biotechnologies, 1722 Aston University, Aston Triangle, Birmingham B4 7ET, U.K.; ⊥ Aston Institute for Membrane Excellence, Aston University, Aston Triangle, Birmingham B4 7ET, U.K.; # Department of Biochemistry, School of Science and Technology, 124688 Nottingham Trent University, Nottingham NG11 8NS, U.K.; ¶ Institut de Chimie et des Matériaux Paris-Est (ICMPE), 129409 CNRS, University Paris Est Créteil, UMR 7182, 2 Rue Henri Dunant, Thiais 94320, France; ∇ Department of Chemistry, Sapienza University of Rome, Piazzale A. Moro 5, Rome 00185, Italy; ○ Department of Chemical and Environmental Engineering, Faculty of Engineering, University of Nottingham, University Park, Nottingham NG7 2RD, U.K.

## Abstract

Recent studies have highlighted the limitations of conventional high degrees of PEGylation in drug delivery systems, including immune recognition and reduced efficacy. Approaches such as poly­(ethylene glycol) (PEG) isomerization and shortening of PEG chains have emerged as strategies to mitigate anti-PEG immune responses while preserving key physicochemical properties required for drug delivery. Inspired by these advancements, this study aims to enzymatically synthesize new hybrid polymers incorporating a limited fraction of PEG and biosourced polyols, such as glycerol and diglycerol, as the hydrophilic counterpart, minimizing the amount of PEG by 50% (compared to our previous work). These novel adipate-based tetrapolymers, generated using four different starting materials, outperformed previous systems, offering a tunable and sustainable design for nanomedicine. By strategically limiting the PEG fraction, we preserved the functional benefits of PEGylation, including stealth and amphiphilicity, while advancing toward greener chemistry. The resulting biodegradable PEGylated polyesters were formulated from film rehydration of solid dispersions and increased the water solubility of the model drug curcumin via direct encapsulation of the compound in polymeric nanoparticles. The best performing polymer variant consisted of diglycerol, 1,6-hexanediol, and PEG combined with divinyl adipate (PEGDGA-Hex 50%). Its drug interactions, colloidal stability, biodegradability, and biocompatibility, in both in vitro (Caco2, human intestinal epithelial cells MCF-7, human breast cancer cells, and MDA-MB-231 late-stage triple-negative breast cancer cells) and invertebrate in vivo models that align with 3R principles (*Caenorhabditis elegans* and *Drosophila melanogaster*), support its potential use in systemic drug delivery.

## Graphical abstract

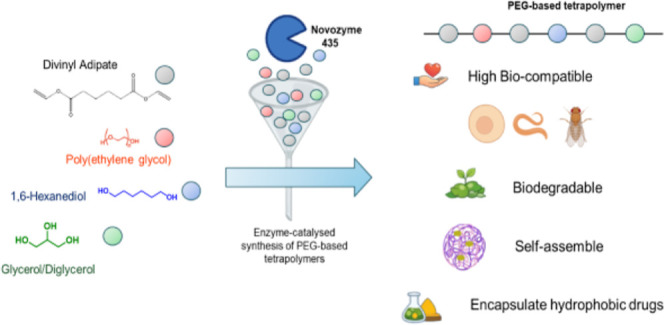

## Introduction

Drugs in the development pipeline have a number of formulation challenges, including low bioavailability, stability issues, toxicity concerns, scalability, and manufacturability challenges.^1–3^ One of the major limitations, though, remains the poor water solubility.^3,5^ Low solubility leads to low or slow absorption in vivo, resulting in poor bioavailability.^3,5^ Approximately 40% of currently marketed drugs and nearly 90% of new drug candidates suffer from low water solubility, leading to inadequate dissolution, poor bioavailability, and suboptimal therapeutic outcomes^3–6^ .

**Scheme 1:**
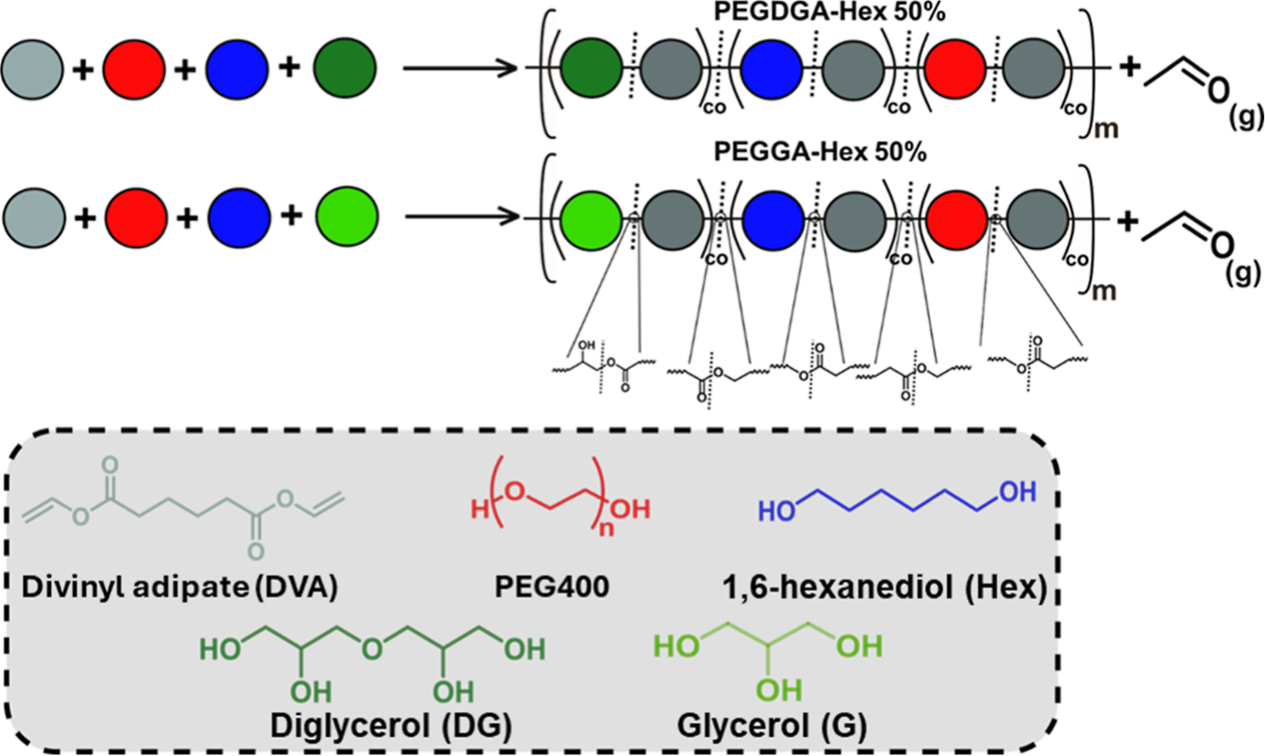
Enzymatic Synthesis of the New Library of PEG-Based Tetrapolymers^a^

a The synthesis is based on the minimization of the amount of PEG by the incorporation of glycerol or diglycerol. Dashed lines indicate that the diols in the polyester backbones may not be ideally alternating. *­(g) Stands for gaseous product. *In the final polymer acronyms, **DVA** becomes **A,** as it represents the adipic part in the polymer.

To address the challenge of poor drug solubility and enhance bioavailability, researchers and pharmaceutical companies have investigated a variety of approaches, including either formulation strategies or solubilization techniques, using cosolvents, surfactants, and cyclodextrins as inclusion complexes to enhance the solubility of drugs in aqueous environments,^3,7^ or salt formation to facilitate better dissolution and absorption.^3^ Solid dispersions (SDs) have been widely recognized as an effective formulation strategy for improving the solubility, dissolution rate, and bioavailability of poorly water-soluble drugs.^8–12^ SDs typically function by dispersing the drug in a polymer matrix, thereby enhancing its wettability and surface area for dissolution.^13,14^ In many cases, the drug may also be converted to a partially or fully amorphous state, which reduces crystallinity and increases dissolution potential.^15,16^ SDs can be prepared using a number of techniques, selected based on the physicochemical properties of the drug and carrier, as well as the desired characteristics of the final formulation.^17–20^ Common approaches include solvent evaporation,^11,12,19,21^ hot melt extrusion,^22^ spray drying,^23–25^ freeze-drying, and coprecipitation.^25–29^ A model system to investigate polymer–drug interactions is solvent evaporation, where the drug and polymer are codissolved and the solvent is removed to yield the solid matrix.^11,12,19,21^ Upon dispersion in aqueous media, SDs can promote the formation of supersaturated solutions, with the polymer stabilizing drug-rich nanodroplets that act as reservoirs. These nanodroplets sustain elevated concentrations of the free drug, improving its supersaturation and facilitating improved permeation at the absorption site and enhancing overall systemic availability.^30,31^ Moreover, upon dissolution in aqueous media, SDs generate transient drug-rich nanoparticles (NPs), whose stabilized nanostructures play a key role in sustaining and enhancing supersaturation throughout dissolution.^32^

In parallel, drug solubility and, therefore, drug delivery can also be enhanced through encapsulation into polymeric NPs.^33–37^ These carriers can be prepared by several established techniques, including nanoprecipitation,^38^ nanoemulsion-based methods,^39–42^ and film rehydration.^29,43–45^ Notably, film rehydration is widely employed in clinically approved nanomedicines such as Genexol and various vaccine formulations,^46^ as well as in the preparation of lipid-based NPs.^47^ Conceptually, this approach shares multiple features with SD formation via solvent evaporation, particularly in the generation of nanoscale drug–polymer assemblies upon rehydration.

Polymers play a critical role in the formulation of both solid SDs and NPs, acting as carriers that increase interaction with drugs, minimizing drug–drug interactions and improving both solubility and bioavailability.^48^ Among the various polymers used in SDs, poly­(ethylene glycol) (PEG) stands out due to its biocompatibility,^43^ high aqueous solubility allowing for improved drug stability,^44^ solubility, and reduced clearance rates.^49–52^

By shielding the drug from enzymatic degradation and other forms of degradation in the body, such as hydrolysis, their pharmacokinetics are modified, and drug efficacy is improved.^53^ This can reduce the patient dosing frequency and consequently enhance patient compliance.^51,54^ This has led to the production of marketed products using PEG-based amorphous SD medicines, including Gris-PEG approved in 1962 ^55^ and Fenoflide approved in 1993.^56,57^ Currently, there are several FDA-approved products that use pegylated NPs, with the most prominent examples being the mRNA COVID-19 vaccines, which utilize a lipid NP (LNP) system that is surface-pegylated to ensure stability and prolonged circulation in the bloodstream.^58^

While PEG is a common excipient in SDs, its drawbacks include the potential for slow dissolution and disintegration of tablets, especially with lower-molecular-weight PEGs. Additionally, any additional surfactants must be carefully selected to avoid interactions that could hinder the drug dissolution.^59,60^ Owing to its hydrophilic nature,^61^ PEG can also face challenges in forming stable SDs with hydrophobic drugs, due to the tendency of hydrophobic drugs to crystallize and phase-separate from the polymer matrix.^62,63^ Furthermore, PEG is nonbiodegradable (at high molar masses), which raises toxicity concerns as it has been shown to bioaccumulate over time, leading to adverse reactions.^50,64,65^

We previously synthesized PEG-based polyesters, harnessing the advantages of PEG, including stealth and hydrophilicity while incorporating ester linkages between PEG groups to facilitate hydrolytic degradability.^61^ Divinyl adipate (DVA) was enzymatically polymerized with PEG to form the amphiphilic copolymer poly­(polyethylene glycol adipate) (PEGA). The enzymatic synthesis used Novozym 435, an immobilized lipase resin, allowing for a controlled polycondensation reaction at mild conditions. Furthermore, the ease of separation and robust nature of the immobilized enzyme made it ideally suited to this reaction.^61,66–69^ We also demonstrated that the incorporation of hydrophobic diols, including 1,6-hexanediol, into the PEGylated backbone significantly improves NP formation, stability, and drug encapsulation.^79^

The present study expands on the potential to develop hybrid polymers, incorporating a limited amount of PEG, while utilizing bioderived polyols such as glycerol, in combination with 1,6-hexanediol, to synthesize fine-tuned polymers that are highly suited to drug delivery applications.^69–71^ This study examines the use of these biodegradable and biocompatible polymers as polymeric nanocarriers for SD, eliminating the need to combine the polymers with a PEGylated surfactant, further reducing dependency on highly PEGylated systems.^72^

## Materials and Methods

### Materials

All chemicals were used as received without further purification unless otherwise stated. DVA was purchased from Tokyo Chemical Industries, UK. PEG400, glycerol, diglycerol, and 1,6-hexanediol (Hex) were purchased from Sigma-Aldrich, UK. Phosphate-buffered saline (PBS), bovine serum albumin (BSA), curcumin, Novozym 435 lipase, derived from *Candida antarctica* immobilized on an acrylic macroporous resin, and lipase from porcine pancreas were also purchased from Sigma-Aldrich. Tetrahydrofuran was purchased from Fisher Scientific UK, and acetone and acetone-*d*_6_ were purchased from Sigma-Aldrich. Matrix-assisted laser desorption/ionization (MALDI) matrix *trans*-2-[3-(4-*tert*-butylphenyl)-2-methyl-2-propenylidene]­malononitrile (DCTB) (98%) was purchased from Sigma-Aldrich and was recrystallized in ethanol. Sodium trifluoroacetate (98%) was purchased from Thermo Scientific.

### Characterization

#### NMR Spectroscopy

Successful polymer synthesis was confirmed using nuclear magnetic resonance (NMR) spectroscopy. NMR spectra [^1^H NMR (16 scans), ^13^C NMR (4096 scans), multiplicity-edited HSQC, HMBC, COSY, and DOSY] were recorded using a Bruker AVANCE 400 or 500 MHz spectrometer. DOSY data were acquired using a dstebpgp3s convection-compensated pulse sequence at 298 K in acetone-*d*_6_, with a diffusion delay of 200 ms and sinusoidal gradient pulses of 2 ms. 32 gradient increments were recorded with a pulse width of 10 μs, acquisition time of 1.59 s, and relaxation delay of 4 s. Diffusion coefficients were obtained using a monoexponential fit. Chemical shifts are given in ppm. Approximately 40 mg of polymer was dissolved in 0.7 mL of solvent. NMR spectra were referenced to 2.05 ppm for ^1^H NMR spectroscopy and 29.84 ppm for ^13^C NMR spectroscopy in acetone-*d*_6_. MestReNova 15.0.0 copyright 2023 (Mestrelab Research S.L.) was used for analysis.

### MALDI Mass Spectrometry

A Bruker UltrafleXtreme MALDI time-of-flight mass spectrometer was used to investigate the structure of four-component polyesters. The matrix DCTB and samples were dissolved in THF with a concentration of 10 mg mL^–1^ and the ionization agent sodium trifluoroacetate with a concentration of 1 mg mL^–1^. Matrix, sample, and salt solutions were mixed in a ratio of 100:10:1. 0.5 μL of this mixture was spotted onto the MALDI target via the dried droplet method. Spectra were recorded in the reflectron mode in a mass range of 400–4000 *m*/*z* and calibrated with PEG standards from Fluka (600 and 1500 g mol^–1^). Bruker FlexAnalysis Software 3.0 was used for processing the obtained spectra, and peak assignments and data interpretation were assisted by the MALDI interpretation tool, MALINTO.^73^

### Gel Permeation Chromatography

Polymer number–average molar mass (*M*_n_) and dispersity (*Đ*) were determined using gel permeation chromatography (GPC) in THF (HPLC grade) eluent at 40 °C. Chromatographs were recorded using two Agilent PL-gel mixed D and two E columns in series with a flow rate of 1 mL min^–1^ and an injection loop of 50 μL. Samples were detected using a differential refractometer. Samples were prepared by dissolving the sample (6 mg) in THF (2 mL) and filtering through a 0.22 μm Teflon filter. Low dispersity poly­(methyl methacrylate) standards were used for the system calibration with average molar masses ranging from 540 to 1.02 × 10^6^ g mol^–1^.

### Differential Scanning Calorimetry

Thermal properties of the polymers were determined using differential scanning calorimetry (DSC). Analysis was performed on a TA-Q2000 (TA Instruments), calibrated with sapphire and indium standards under N_2_ flow at 50 mL min^–1^. The polymer (∼5 mg) was weighted into a T-zero aluminum pan (TA Instruments), with a reference pan (T-zero aluminum) remaining empty. Pan lids were pin-holed, small amounts of residual solvent were in the polymer, and samples were heated at a rate of 10 °C min from −90 to 200 °C. Two heating cycles were recorded in order to remove any thermal history of the polymers. The second heating cycle was used to determine the glass transition temperature (*T*_g_) and melting temperature (*T*_m_) of polymers.

### Water Contact Angle

Water contact angle (Θ_w_) samples were prepared by solvent casting of the polymer from a solution in acetone onto a microscope glass coverslip. Samples were prepared at a concentration of 3 mg mL^–1^, by pipetting 3–4 drops of polymer solution onto the whole surface of the coverslip and letting the solvent evaporate overnight to produce a thin film of polymer. Images were captured using an iPhone camera and were analyzed using the ImageJ software using a drop angle-LB-ADSA extension to measure the water contact angles. Samples were measured at ambient temperature with at least three replicates of each measurement recorded at two different time points, *t* = 0 (the exact moment when water touches the polymer surface) and *t* = 5 s (as soon as the drop settles on the polymer surface).

### Polymer Synthesis

The tetrapolymers (Scheme 1) were synthesized similarly to previously published protocols for the polycondensation of PGA-Hex.^61,69,71^ DVA (2.48 g, 12.50 mmol) and the corresponding amounts of PEG, glycerol or diglycerol, and 1,6-hexanediol were weighed into a 20 mL glass vial and were subsequently dissolved in THF (10 mL) at 50 °C. Novozym 435 (0.11 g) was added to the reaction once at 50 °C, and the reaction was stirred for 5 h. The reaction vial was sealed with a rubber septum and pierced with a needle to allow the removal of acetaldehyde. After 5 h, the reaction was stopped by removing the enzyme by gravity filtration. The solvent was removed in vacuo, affording a viscous yellow liquid.

The ratio between glycerol/diglycerol and 1,6-hexanediol was 50:50; therefore, the polymers are labeled 50%, similar to our previous work.^61^ Two different ratios between the hydrophilic diols, PEG/glycerol/diglycerol, were investigated, 1:1 and 4:1. The amounts of each reagent used in the synthesis of each polymer have been summarized in Table 1.

**Table 1:** Details of Reagents Used in Polymer Synthesis^a^ ^b^

polymer name	DVA (mmol)	DVA (g)	glycerol (mmol)	glycerol (g)	diglycerol (mmol)	diglycerol (g)	PEG (mmol)	PEG (g)	1,6-hexanediol (mmol)	1,6-hexanediol (g)
PEGA-Hex 50%	12.50	2.48	N/A	N/A	N/A	N/A	6.25	2.50	6.25	0.74
PEGGA-Hex 50%1:1	12.50	2.48	3.13	0.29	N/A	N/A	3.13	1.25	6.25	0.74
PEGGA-Hex 50%4:1	12.50	2.48	1.25	0.12	N/A	N/A	5.00	2.00	6.25	0.74
PEGDGA-Hex 50%1:1	12.50	2.48	N/A	N/A	3.13	0.52	3.13	1.25	6.25	0.74
PEGDGA-Hex 50%4:1	12.50	2.48	N/A	N/A	1.25	0.21	5.00	2.00	6.25	0.74

a PEGA-Hex 50% was used as the control and has been previously synthesized.^61^

b In the final polymer acronyms, **DVA** becomes **A**, as it represents the adipic part in the polymer.

For example, for the polymer entitled PEGDGA-Hex, 50% 1:1, % refers to the amount of 1,6-hexanediol and 1:1 is the ratio between the hydrophilic diols. The moles of the monomers are adjusted according to moles_hydrophilic monomers_ + moles_hexanediol_ = 12.5 mmol = moles_DVA_.

### NP Formulation and Characterization

#### NP Formulation

The self-assembly behavior of the PEG-based tetrapolymers into NPs and their ability to encapsulate poorly soluble drugs were evaluated via film rehydration of free polymers or SD with a model drug formulation. Briefly, 10 mg of polymer was dissolved in 1 mL of acetone, and the solution was left to evaporate overnight under ambient conditions. The resulting solid residues were subsequently resuspended in 4 mL of deionized (DI) water to achieve a final polymer concentration of 2.5 mg mL^–1^.

### Dynamic Light Scattering Measurements

Particle size and zeta potential were measured using a Zetasizer Nano ZS spectrometer (Malvern Instruments Ltd., UK). Experiments were performed with a 633 nm laser at a fixed angle of 173°. Samples were equilibrated for 30 s at 25 °C prior to measurement. All samples were measured in triplicate. NPs were prepared at a concentration of 2.5 mg mL^–1^ and filtered through a 0.22 μm cellulose filter prior to analysis.

### Curcumin Encapsulation Analysis

Dye/polymer solutions were prepared as follows:

A solution of curcumin in acetone was prepared at a concentration of 0.5 mg mL^–1^. The polymer (10 mg) was weighed directly into a clean glass vial, and 1 mL of curcumin solution was added (polymer/drug ratio of 20:1 w/w). Polymer solutions were left overnight to enable acetone evaporation and later resuspended using DI water (4 mL). NP-drug dispersions from SD were filtered through a 0.22 μm filter. Dye controls containing the drug in water without the addition of the polymer were filtered using the same filters. Particle size and zeta potential were then measured.

Encapsulation of curcumin was qualitatively determined using fluorescence spectrophotometry, by measuring the fluorescence intensity of the NP-dye dispersions at λ_ex_ = 467 nm and λ_em_ = 550 nm for curcumin. The apparent water solubility enhancement of the dyes formulated with our polymers was semiquantitatively evaluated using eq 1.

1 $$\Delta F\%=\frac{\Delta F}{F}=\frac{\left(F_{NPs}-F_{DYE}\right)}{F_{DYE}}\times 100$$

Equation 1: Calculation of the apparent change in fluorescence intensity of the encapsulated curcumin; this value can be correlated to the increase in the apparent solubility of the dyes.

*F*_NPs_ = fluorescence signal of NP formulation with encapsulated dye, normalized by the polymer *F*, and *F*_DYE_ = fluorescence signal of the free dye in water.

### NP Stability in BSA

Stock solutions of NPs (with and without drug) were prepared at 2.5 mg mL^–1^ in DI water. A stock solution of BSA was prepared at 2 mg mL^–1^ in DI water, following the published protocol.^69^ NP stock solution (100 μL) was mixed with BSA (100 μL) in a well plate. Samples were measured in the Zetasizer at 0, 2, and 24 h to assess the stability of NPs.

### Transmission Electron Microscopy Measurements

Dry-state transmission electron microscopy (TEM) imaging was conducted by using an FEI TECNAI F20 microscope with an acceleration voltage of 200 kV. Aqueous suspension samples were applied onto Formvar-graphene oxide-coated copper grids (400 mesh). 5 μL of the samples was deposited onto the grid. After an incubation period of approximately 90 s, the excess sample was removed by blotting from the grid. Following this, the grid underwent staining with an aqueous uranyl acetate (UA) solution (1 wt %) for 90 s before being subjected to blotting, drying, and subsequent microscopic analysis. UA is used as a negative staining agent to enhance the contrast of polymeric NPs in TEM imaging. ImageJ was employed for image processing.

### Small-Angle X-ray Scattering Measurements

Small-angle X-ray scattering (SAXS) patterns were recorded at a synchrotron source (Diamond Light Source, beamline I22,^74^ Didcot, UK; Experiment ID SM38357-1) using monochromatic X-ray radiation (X-ray wavelength λ = 1.00 Å, with scattering vector q ranging from 0.0017 to 0.17 Å-1, where *q* = 4π sin θ/λ and θ is one-half of the scattering angle) and a 2D Pilatus 2 M pixel detector (Dectris, Switzerland). All static SAXS measurements were performed on 2.5 mg mL^–1^ copolymer dispersions in 1.55 mm internal diameter glass capillaries. Scattering data were reduced and normalized utilizing standard routines available at the beamline.^75,76^

### Enzymatic Degradation/Hydrolytic Enzymatic Assay

Lipase from porcine pancreas, type II [≥125 units/mg protein (using olive oil (30 min incubation)], 30–90 units/mg protein (using triacetin), was used in this experiment. A solution of the enzyme at 10 mg mL^–1^ in PBS was prepared. 50 μL of this solution was added to 200 μL of NPs (at a concentration of 2.5 mg mL^–1^ in water, as mentioned previously). The effect of the enzyme was observed within 24 h at 25 °C.

### Cytotoxicity Study

#### In Vitro Cell Culture

The human epithelial cell line Caco-2, the MCF-7 human breast cancer cells, and the MDA-MB-231 late-stage triple-negative breast cancer cells were obtained from the American Type Culture Collection (ATCC) and used across a 10-passage window. Cells were cultured in Dulbecco’s modified Eagle medium (DMEM) supplemented with 10% fetal bovine serum (FBS) at 37 °C in a humidified incubator with 5% CO2. Cells were routinely grown in 75 cm^2^ culture flasks to 70% confluence.

### In Vitro PrestoBlue Metabolic Activity Assay

Cellular metabolic activity was measured using the PrestoBlue viability assay (Thermo Fisher Scientific) as an indication of cytotoxicity. Caco-2, MCF-7, and MDA-MB-231 cells were seeded at 1 × 10^4^ cells per well in 96-well plates and cultured for 24 h prior to assaying. Cells were exposed to treatments in 100 μL phenol red-free DMEM containing 10% FBS for 24 h. Triton X-100 was applied at 1% (v/v) as a positive cell death control, and medium alone was used as a negative control. Following the exposure period, treatments were removed and cells incubated with 100 μL 10% (v/v) PrestoBlue reagent per well and diluted in phenol red free medium for 60 min. The resulting fluorescence was measured on a Tecan Spark 10 M plate reader at λ_ex_ = 560 nm and λ_em_ = 600 nm. Relative metabolic activity is calculated from PrestoBlue data by setting values from the negative control as 100% and positive control values as 0% metabolic activity (eq 2).

2 $$Relative\;metabolic\;activity=\left(\frac{\left(x-positive\;control\right)}{\left(negative\;control-positive\;control\right)}\right)\times 100$$

Equation 2: Calculation of relative metabolic activity. *x* = treated sample fluorescence value. All values are from fluorescence at 560/600 nm (λ_ex_/λ_em_).

### In Vitro Lactose Dehydrogenase Release Test

To study plasma membrane damage in vitro, the extracellular release of lactose dehydrogenase (LDH) enzyme was assessed using the LDH release assay (Sigma-Aldrich, TOX7 kit). As given above, Caco-2, MCF-7, and MDA-MB-231 cells were seeded at 1 × 10^4^ cells per well in 96-well plates and cultured for 24 h. Cells were exposed to treatments in 100 μL phenol red-free DMEM containing 10% FBS and received either polymeric formulations, 1% Triton X-100 to induce cell lysis, or DMEM only to serve as the vehicle control. Following 24 h exposure, 50 μL of supernatant per well was sampled and transferred to a new 96-well plate for the detection of LDH released extracellularly. LDH detection solution was prepared according to the manufacturer’s instructions, and 100 μL of LDH detection reagent was added per well to the 50 μL of supernatant sample. The solution was incubated at room temperature protected from light, for 25 min, and the absorbance of the resulting solution was measured at 490 nm on a Tecan Spark 10 M plate reader. Relative LDH release was calculated by setting the absorbance signal of 1% Triton X-100, assumed to generate full cell lysis, as 100% LDH release and the background signal generated by LDH detection solution alone as 0%.

### Biocompatibility Studies

#### In Vivo Assessment Using *C.elegans*

Synchronized *Caenorhabditis elegans* (*C. elegans*) embryos were allowed to develop to the young adult stage by incubating for 3 days (20 °C) on nematode growth medium with an *Escherichia coli* (*E. coli*) OP50 lawn. The nematodes were collected with M9 buffer [(KH_2_PO_4_) (0.022 M), Na_2_HPO_4_ (0.042 M), NaCl (0.086 M), and MgSO_4_ (1 mM)], filtered (Merck 60 μm Nylon net), and washed with M9 buffer to obtain young adults on a filter.

Nematodes were counted and adjusted to 20–30 nematodes in polymeric NPs (0.5 mg mL^–1^), which were suspended in M9 buffer solution containing *E. coli* OP50 (0.2 OD_600_) for sustenance. *E. coli* OP50 alone was used as a viable (positive) control and ethanol (20% v/v) as a nonviable (negative) control. All treatments were conducted in triplicate. Images were taken with an EVOS FL microscope (Thermo Fisher Scientific, UK) after 24 h incubation with a 4× objective (0.13 NA). Viability was evaluated as a function of motility.^77^

### In Vivo Assessment Using *Drosophila melanogaster*

#### Drosophila Stocks

All experiments were conducted using the *D. melanogaster* strain w^1118^ (Bloomington Drosophila Stock Center, BDSC #2376, Bloomington, IN). Flies were maintained on NutriFly medium (Genesee Scientific), a standardized cornmeal-based diet formulated to support consistent nutritional quality across developmental and aging assays. Stocks were kept at 25 °C, 60–70% relative humidity, under a 12:12 h light–dark cycle. These conditions were routinely used to ensure robust circadian entrainment and minimize environmental variability.

### Fly Food Preparation and Drug Supplementation

NutriFly medium (Genesee Scientific) was prepared according to the manufacturer’s instructions.^78^ Drug-supplemented media were prepared to yield final concentrations of 0 μg/mL (control), 10 μg/mL, 100 μg/mL, and 1000 μg/mL of the test compound. Briefly, 1 mL of 30 mg/mL, 3 mg/mL, or 0.3 mg/mL polymer solutions was mixed with 29 mL of food to generate the 1000 μg/mL, 100 μg/mL, and 10 μg/mL preparations, respectively. After thorough mixing, 5 mL aliquots were dispensed into 24 vials (six vials per dose; three biological replicates for each sex). Vials were allowed to cool and solidify at room temperature and were subsequently stored under conditions that prevented desiccation.

### Drosophila Cross Setup

An equal number of males and females were dispensed into the drug-treated and control food vials at 23°. F1 progeny were collected within a 24 h eclosion window to generate age-synchronized experimental cohorts. When sex-specific analyses were required, male and female flies were separated immediately upon eclosion to prevent unintended mating and maintain single-sex cohorts. This procedure followed established protocols routinely applied in Drosophila neurobiology and aging research.^79^

### Longevity and Climbing Assay

Lifespan analysis was conducted using standard protocols.^80,81^ Briefly, 30 flies were put in each vial, and survival was monitored for a period of 6 weeks. Flies were transferred to fresh medium at regular intervals to preserve the food quality and prevent microbial contamination. Mortality was recorded at each transfer, and individuals lost due to handling or escape were censored from subsequent survival analyses, in accordance with standard practice. Each lifespan assay included *n* = 3 biological replicates, with each replicate representing an independently established cohort maintained in parallel under identical conditions.

Negative geotaxis behavior was assessed using a Drosophila climbing assay following standard protocols. For each treatment condition, 30 age-matched adult male flies were transferred into a clean 15 cm plastic vial with the finish line marked vertically at 10 cm. Flies were gently tapped to the bottom of the cylinder, and their climbing performance was recorded over a 20 s interval. The number of flies that climbed above the marked finish line within 20 s was quantified and expressed as the percentage of the successful climbers. Three independent biological replicates were performed for each treatment group to ensure reproducibility.

### Statistical Analysis

All experiments were performed in triplicate, and analysis was performed using GraphPad Prism software (v10). Cytotoxicity results for in vitro cell culture were tested for significant differences from the control group (DMEM) using two-way ANOVA and Dunnett’s multiple comparisons posthoc test with statistical significance determined at *P* < 0.05. Survival data were analyzed using the Kaplan–Meier estimator, and differences between survival curves across multiple treatment groups were assessed using log-rank (Mantel–Cox) tests with appropriate multiple-comparison procedures. Climbing assay data between different groups were analyzed by two-way RM ANOVA using GraphPad Prism.

## Results and Discussion

Enzymatically synthesized PEG-based polyesters can enhance the biocompatibility while mitigating some of their inherent limitations, including excessive hydrophilicity, restricted capacity to encapsulate hydrophobic drugs, and concerns regarding persistence and toxicity.^61^ In particular, from our polymeric library, PEGA-Hex 50%, generated by polycondensation of DVA, PEG, and 1–6-hexanediol at a 50:50 ratio between PEG/1,6-hexanediol, emerged as the most promising candidate, combining favorable self-assembly into stable NPs with the practical advantage of readily accessible starting materials. Building on this foundation, the present study employs enzymatic synthesis of polyesters in a similar manner using PEG, DVA, and hexanediol but introduces glycerol and diglycerol as additional bioderived hydrophiles. Using glycerol and diglycerol, the PEG content of the polyesters can be further reduced, thereby enhancing the sustainability and green-chemistry profile of these polymers. We present four tetrapolymers, comprising two hydrophilic-diol ratios (PEG/glycerol/diglycerol = 1:1 and 4:1), each combined with a fixed 50:50 hydrophilic-to-hydrophobic diol ratio. This strategy investigates the influence of polymer amphiphilicity on NP formation, aiming to promote more efficient self-assembly into biodegradable NPs. The self-assembling properties of the tetrapolymer library were assessed by both nanoprecipitation and SD.

### Synthesis and Physiochemical Characterization of PEG-Based Tetrapolymers

Enzymatic polycondensation was carried out under mild conditions using *C. antarctica* lipase B (CalB), immobilized acrylic resin lipase, commercially known as Novozym 435, enabling a regio- and chemo-controlled step-growth polymerization reaction.^82,83^ The successful formation of the polymer was confirmed through a series of analytical techniques. ^1^H NMR spectroscopy demonstrated the consumption of the DVA monomer by the disappearance of its characteristic vinyl proton signals at 4.59, 4.87, and 7.29 ppm (Figures 1A and S1).^84^ Concurrently, the appearance of new resonances at 3.60 and 4.20 ppm, attributed to the –OCH_2_–CH_2_– protons of the PEG unit adjacent to the ester linkage, indicated successful polymer formation.^61^ These assignments were further validated by HSQC and HMBC 2D-NMR analysis, which confirmed the successful esterification of the alcohol monomers during the polymerization process (Figures S3–S6). In addition, the successful transesterification of DVA was confirmed by the presence of two broad peaks at 2.33 and 1.63 ppm, indicative of carbonyl adjacent to a –CH_2_ peak and an alkyl –CH_2_ peak, respectively (Figures 1A and S1). As previously reported,^68^ protons corresponding to glyceride moieties appeared at 4.40 ppm, consistent with esterification of the glycerol hydroxyl groups. Moreover, the signals associated with glycerol and the adipate ester exhibited downfield shifts and peak broadening relative to those of the monomers, collectively confirming successful polymerization. For the diglycerol variant, a broadening of the −CH_2_ proton resonance at 3.51 ppm, attributed to ether-adjacent protons of diglycerol, was observed. Upon esterification, a downfield shift of these −CH_2_ protons to 4.11 ppm was detected, consistent with successful formation of ester linkages. Additionally, a broad signal at 3.97 ppm was assigned to the methine proton of the 1,3-disubstituted diglycerol unit. Diffusion-ordered NMR spectroscopy (DOSY) analysis demonstrated that all ^1^H NMR peaks corresponding to moieties within the polymer backbone exhibited very similar diffusion coefficients. This indicates that all four monomers were successfully incorporated into one polymer (Figure 1B). In addition, the ratio between the monomers was confirmed by ^1^H NMR analysis, in agreement with the expected composition (Figure S2).

**Figure 1:**
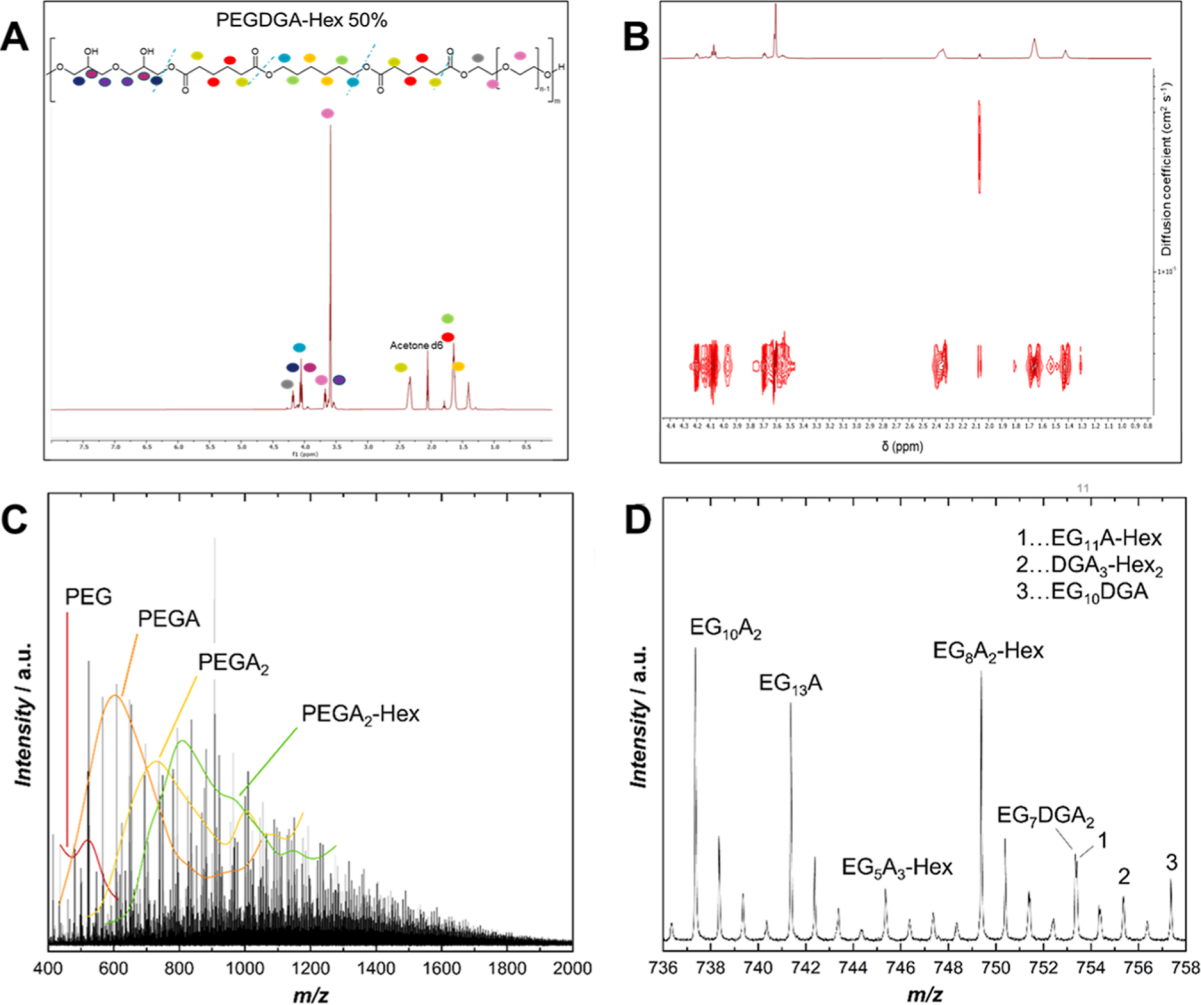
Physicochemical characterization results for PEGDGA-Hex 50%. (A) ^1^H NMR spectra (polymer structure is reported with key chemical features and not in full to maximize space). (B) DOSY NMR spectrum of PEGDGA-Hex 50% 1:1. (C) MALDI mass spectrum of PEGDGA-Hex 50% 1:1, including different PEG homopolymer series. (D) MALDI-ToF mass spectrum confirming the reaction of all four building blocks: ethylene glycol (EG) from PEG, diglycerol (DG), adipic acid (A), and 1,6-hexanediol (Hex).

The incorporation, in the same polymeric backbone, of all four building blocks was also confirmed by MALDI-ToF mass spectrometry. Figure 1C shows the mass spectrum of PEGDGA-Hex 50% with the highlighted series of PEG homopolymer, PEG reacted with one or two adipic acid units, and the copolyester with hexanediol. Further series, including diglycerols, were observed. However, the peak regions were not suitable for displaying a comparable series because of peak overlap. With increasing molar mass, the number of different comonomer combinations rises exponentially. Several species showing the incorporation of all monomers are highlighted in the small *m*/*z* range shown in Figure 1D, as well as a copolyester without polyethylene glycol (DGA_3_-Hex_2_), demonstrating the complex mixtures present in these polymers.

GPC analysis further proved the successful polymerization, as an increase in *M*_n_ from 330 and *Đ* of 1.25 of free PEG to an *M*_n_ of 3500 to 5400 and *Đ* up to 1.90 was observed for all tetrapolymers (Table 2). The increase dispersities, characteristic of step-growth polymerization, further supported the occurrence of polymerization (Table 2 and Figure S7).

**Table 2:** *M*_n_, *Đ*, *T*_g_, *T*_m,_ and Δ*H*_m_ of the Tetrapolymers

polymer	*M*_n_ (g/mol)^a^	*D̵* ^a^	*T*_m_ (°C)^b^	Δ*H*_m_ (J/g)^b^	*T*_g_ (°C)^b^
PEG400	330	1.25	5.2	107.2	-
PEGA-Hex 50%	3500	1.90	–9.1	26.2	–61.4
PEGDGA-Hex 50% 1:1	4300	1.65	16.7	7.4	–55.9
PEGGA-Hex 50% 1:1	5400	1.32	18.9	5.4	–56.0
PEGDGA-Hex 50% 4:1	4800	1.59	19.2	2.1	–53.4
PEGGA-Hex 50% 4:1	3500	1.26	24.2	4.9	–51.1

a Molecular weight was determined by GPC using THF eluent at 40 °C. GPC was calibrated using PMMA standards and dispersity (*Đ*) of approximately 1.

b Thermal properties were determined by the DSC.

The PEG-based tetrapolymers displayed thermal properties distinct from those of unreacted PEG and the previously reported PEG-based terpolymer PEGA-Hex 50%, which served as a control in this study.^61^ The thermal behavior of the synthesized tetrapolymers further supports their distinct structural characteristics compared to both PEG and the previously reported PEGA-Hex 50% control. DSC analysis demonstrated that PEG exhibits a T_m_ of 5.2 °C and no detectable glass transition within the studied range, consistent with its semicrystalline nature. In contrast, PEGA-Hex 50% showed a markedly reduced melting point (−9.1 °C) and a low T_g_ (−61.4 °C), reflecting increased chain flexibility and disruption of PEG crystallinity due to incorporation of hydrophobic segments.

The newly synthesized tetrapolymers displayed intermediate thermal properties, with melting temperatures ranging from 16 to 24 °C and glass transition temperatures between −51 and −56 °C. The increase in T_m_ compared to PEGA-Hex 50% suggests a partial restoration of ordered domains, likely arising from the introduction of glycerol or diglycerol units, which can promote intermolecular interactions and reduce chain mobility. At the same time, the higher *T*_g_ values compared to PEGA-Hex indicate reduced segmental flexibility, consistent with the presence of multifunctional, more rigid hydrophilic moieties. Variations in enthalpy of melting (Δ*H*_m_) across the series further suggest differences in crystallinity, with lower Δ*H*_m_ values reflecting a predominantly amorphous character, which is advantageous for drug delivery applications such as SDs.

Water contact angles (Θ_w_) of polymeric films were measured to assess the effect of reduced PEG content and the concurrent incorporation of glycerol-based diols on the surface wettability of the resulting polymers. The initial contact angles (*t* = 0) of the PEG-based tetrapolymers were comparable to those of the PEGA-Hex 50% reference material, ranging from 50° to 60°, and were not significantly affected by the inclusion of glycerol or diglycerol, nor by variations in the relative proportions of the hydrophilic diols (Figure 2). All measured values were below 90°, the conventional threshold distinguishing hydrophobic from hydrophilic surfaces, confirming that the materials remained within the hydrophilic regime. However, contact angles clustered around or slightly above 50°, a range often associated with amphiphilic behavior,^85^ suggesting that the polymers exhibit a balanced distribution of hydrophilic and hydrophobic character. Furthermore, water contact angles measured after the water drop settled into all the polymeric surfaces (*t* = 5 s) showed a consistent and statistically significant decrease in value compared to initial measurements, reaching values around 40°. This reduction is attributed to the spontaneous, thermodynamically driven reorientation of hydrophilic segments, namely, PEG, glycerol, and diglycerol, toward the water droplet upon contact, thereby enhancing surface wettability (Figure 2), as is typical of amphiphilic materials. Compared to PEGA-Hex 50%, the tetrapolymers exhibited slightly higher Θ_w_ values, which correlate with the reduced PEG content and the incorporation of shorter hydrophilic glycerol-based diols.

**Figure 2:**
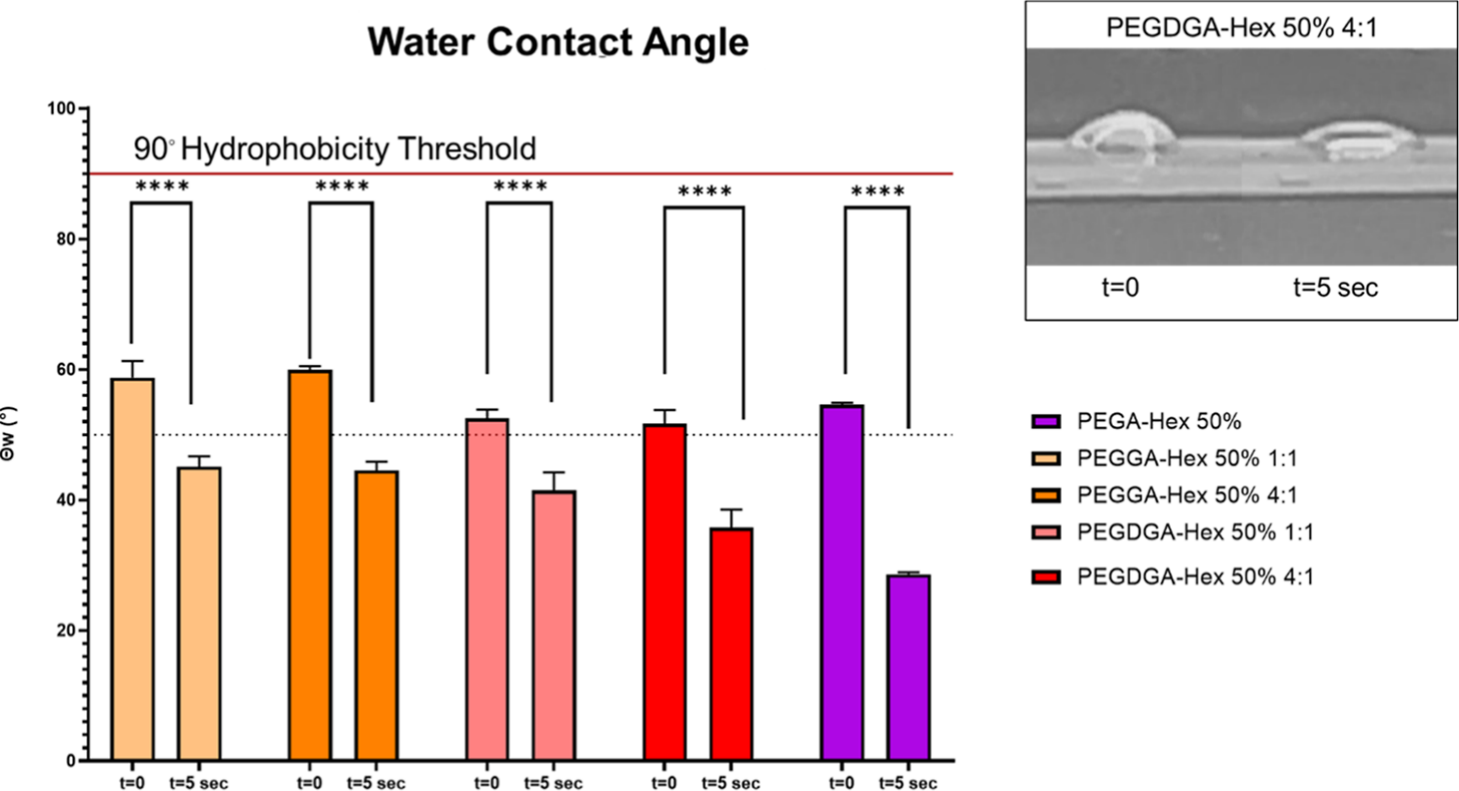
Water contact angle measurements. Measurements were taken at two different time points: *t* = 0 s (as soon as the water droplet touched the polymer surface) and *t* = 5 s (when the water droplet settled on the polymer surface). PEGDGA-Hex 50% 4:1, as the polymer with the highest difference between *t* = 0 and *t* = 5 s, was selected for a close-up comparison of the droplets. Statistical analysis was done using GraphPad Prism software (v10) and a two-way ANOVA comparison, *p* < 0.0001.

### Model Drug Encapsulation Assay

To assess the potential of the newly synthesized tetrapolymers as drug-delivery carriers, an initial encapsulation study was undertaken. This investigation examined the extent to which nanoformulated PEGylated tetrapolymers enhanced the apparent aqueous solubility of a model hydrophobic drug, curcumin, initially using SD films. The performance of these systems was compared with that of PEGA-Hex 50%, which has previously demonstrated effective encapsulation of curcumin.^61^ Curcumin^86^ is particularly well-suited to this qualitative encapsulation study owing to its pronounced water-insolubility, which presents a relevant challenge for evaluating formulation performance. Its intrinsic fluorescence further enables straightforward and sensitive detection, as demonstrated in the previously established protocols.^61,69^ In addition, curcumin exhibits notable biological activities, including anticancer and antimicrobial effects,^87–89^ making it a widely used model compound in drug-delivery research.

This assay served as a preliminary screening tool to differentiate the encapsulation performance of the newly synthesized tetrapolymers and to identify the most promising candidates for further investigation. The percentage change in fluorescence intensity (Δ*F*%) is used as an indirect measure of the improvement in apparent aqueous solubility of the hydrophobic compounds.^69^ Based on eq 1, the synthesized polymers and the controls are ranked quickly and in a semiquantitative way according to their Δ*F*% value (Figure 3A). PEG alone was included as the control material, whereas the glycerol- and diglycerol-based polymers PGA-Hex 50% and PDGA-Hex 50% were excluded from this comparison owing to their inability to form NPs from rehydration of SDs under the conditions employed.

**Figure 3:**
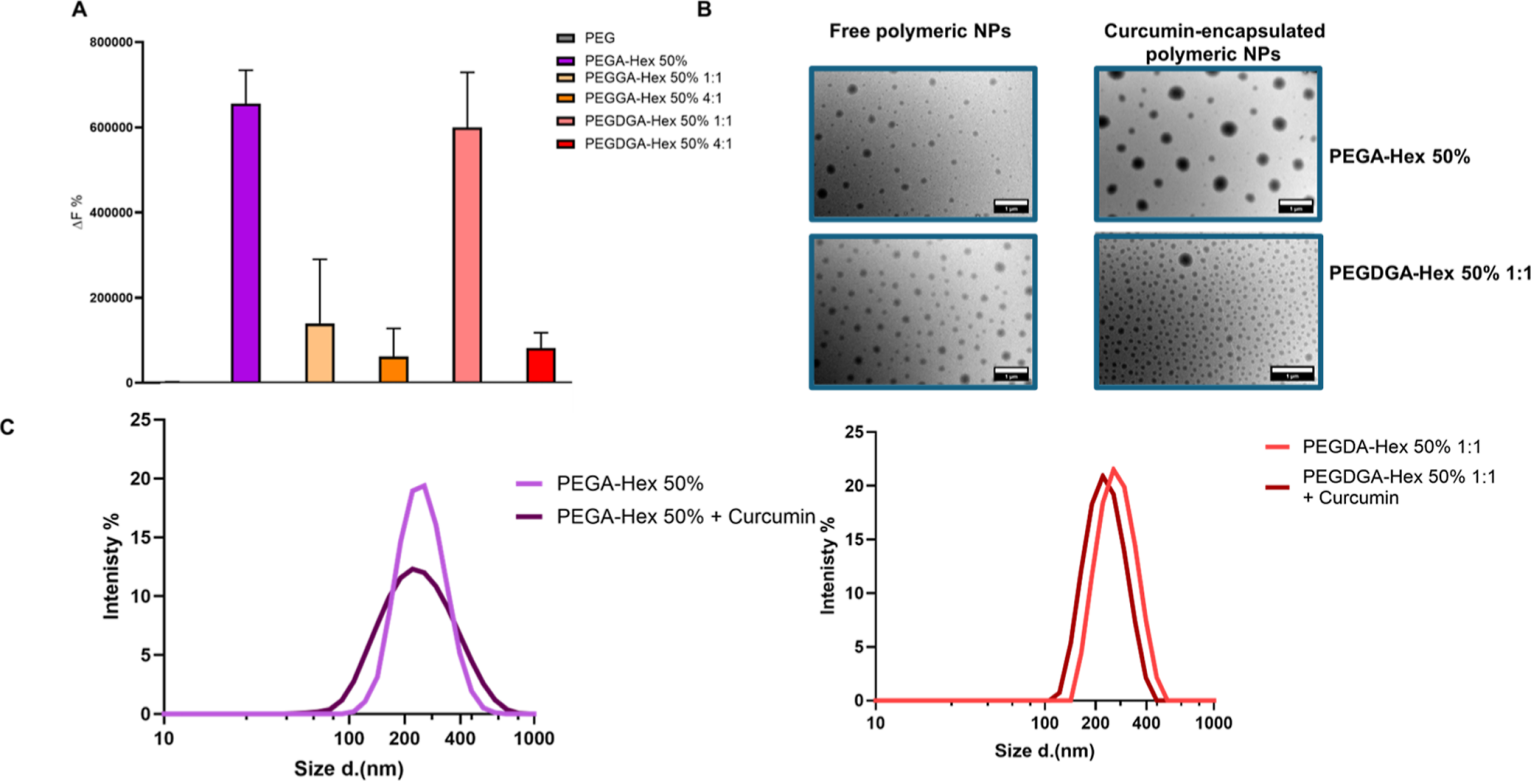
(A) ΔF% ranking of the curcumin-encapsulated tetrapolymeric systems with PEGA-Hex 50% acting as a control. (B) TEM images of the two best-performing polymeric systems; PEGA-Hex 50% and PEGDGA-HEX 50% 1:1 shown as unloaded NPS (B, left) and after encapsulation of curcumin (B, right). All are at a scale bar of 1 μm. (C) DLS traces showing the size distribution of the two best-performing polymeric systems (PEGA-Hex 50% and PEGDGA-HEX 50% 1:1) as unloaded NPs and after encapsulation of curcumin.

Among the tested formulations, PEGA-Hex 50%, serving as the reference system, exhibited the highest Δ*F*% value ([6.6 ± 0.7] × 10^5^), confirming its strong encapsulation capability. PEGDGA-Hex 50% 1:1 displayed a comparable Δ*F*% ([6.0 ± 1.0] × 10^5^), indicating a similar encapsulation efficiency and effective improvement of apparent water solubility of the hydrophobic active ingredient. This suggests that PEGDGA-Hex 50% 1:1 can form stable nanostructures with a high affinity for hydrophobic compound encapsulation when formulated as SD, even when using a minimally PEGylated polymer. This also suggests that the presence of both hydrophobic sections (adipates) and mixed hydrophilic sections (diglycerol and PEG) facilitates different interactions with curcumin, likely hydrophobic, dipole–dipole, and H-bonding interactions. In contrast, the other formulations, including the glycerol variants and PEGDGA-Hex 4:1, exhibited significantly lower Δ*F*% values, indicating reduced encapsulation potential under the same conditions. These results highlight PEGDGA-Hex 50% 1:1 as a promising candidate for hydrophobic drug delivery, comparable in performance to the model with high PEG content.

TEM analysis (Figure 3B) confirms the spherical morphology of the NPs in both polymeric systems. The tetrapolymer PEGDGA-Hex 50% (1:1) and the control PEGA-Hex 50% form NPs of comparable size (218 ± 45 nm and 174 ± 65 nm by TEM, respectively), approximately 200 nm, as measured by DLS.

After encapsulation of the model drug, both systems retain their spherical shape, as observed by TEM. However, while PEGDGA-Hex 50% (1:1) shows no aggregation and only the typical size reduction associated with TEM sample preparation and vacuum treatment (124 ± 19 nm), PEGA-Hex 50% loaded with curcumin exhibits clear aggregation, with TEM sizes centered at 344 ± 93 nm. This behavior may be attributed to the lower stability of the PEGA-Hex system under dry conditions and in the absence of additional intermolecular interactions, which are likely provided by the DG units in the PEGDGA-based system.

DLS analysis was employed to evaluate the influence of drug encapsulation on the hydrodynamic diameter of the model polymer, PEGA-Hex 50%, and the best-performing tetrapolymer, PEGDGA-Hex 50% 1:1. Minimal variations in particle size were observed following the encapsulation of the hydrophobic model compound, indicating preserved colloidal stability of the polymeric assemblies (Figure 3C). The average particle size of PEGDGA-Hex 50% 1:1 remained virtually unchanged (∼200 nm), indicating minimal structural rearrangement and suggesting a high degree of compatibility between the hydrophobic drug and the polymeric matrix (Figure 3C and Table S1). This stable size profile may be due to a robust NP core structure that resists collapse or swelling upon drug incorporation, an essential property for maintaining predictable in vivo behaviors like pharmacokinetics and biodistribution. SAXS results (Figure S9) corroborated the DLS and TEM findings, with both the unloaded control PEGA-Hex 50% and the new tetrapolymer system PEGDGA-Hex 50% 1:1 exhibiting similar scattering profiles. Following curcumin loading, the low-*q* scattering for the PEGA-Hex 50% control increases, indicating the presence of larger NPs as shown in TEM images and with the broadening of the DLS size distribution. For PEGDGA-Hex 50% 1:1, the scattering at low-*q* decreases following curcumin encapsulation and supports DLS and TEM observations that smaller NPs are formed under these conditions. SAXS also revealed that a peak at *q* ≈ 0.12 nm^–1^ is evident when curcumin was loaded within NPs, which could represent the formation of closely interacting particles with a radius of interaction sphere of ∼52 nm.

### NP Stability

The long-term colloidal stability of the NP formulations was assessed over a period of 20 days, using DLS measurements used to monitor changes in hydrodynamic diameter (Figure 4). Interestingly, the NP sizes for PEGDGA-Hex 50% 1:1 remained well within the range of 100–200 nm for the drug, which is broadly considered ideal for drug delivery due to favorable biodistribution, prolonged circulation times, and efficient cellular internalization.^90–92^ Notably, PEGDGA-Hex 50% 1:1 NPs maintained a nearly constant size over the entire 20 day period, indicating high colloidal stability and minimal aggregation (Figure 4B). In contrast, PEGA-Hex 50% NPs showed a significant increase in size after 10 days, suggesting progressive instability likely due to swelling, partial disassembly, or aggregation (Figure 4A). The increased NP stability observed for PEGDGA-Hex 50% 1:1 may be attributed to the structural contributions of diglycerol moieties, which could promote more intra- and intermolecular hydrogen bonding within the polymeric network, enhancing the physical integrity of the NP assemblies. These findings underscore the potential of PEGDGA-Hex 50% 1:1 as a more robust and stable drug delivery carrier over extended periods.

**Figure 4:**
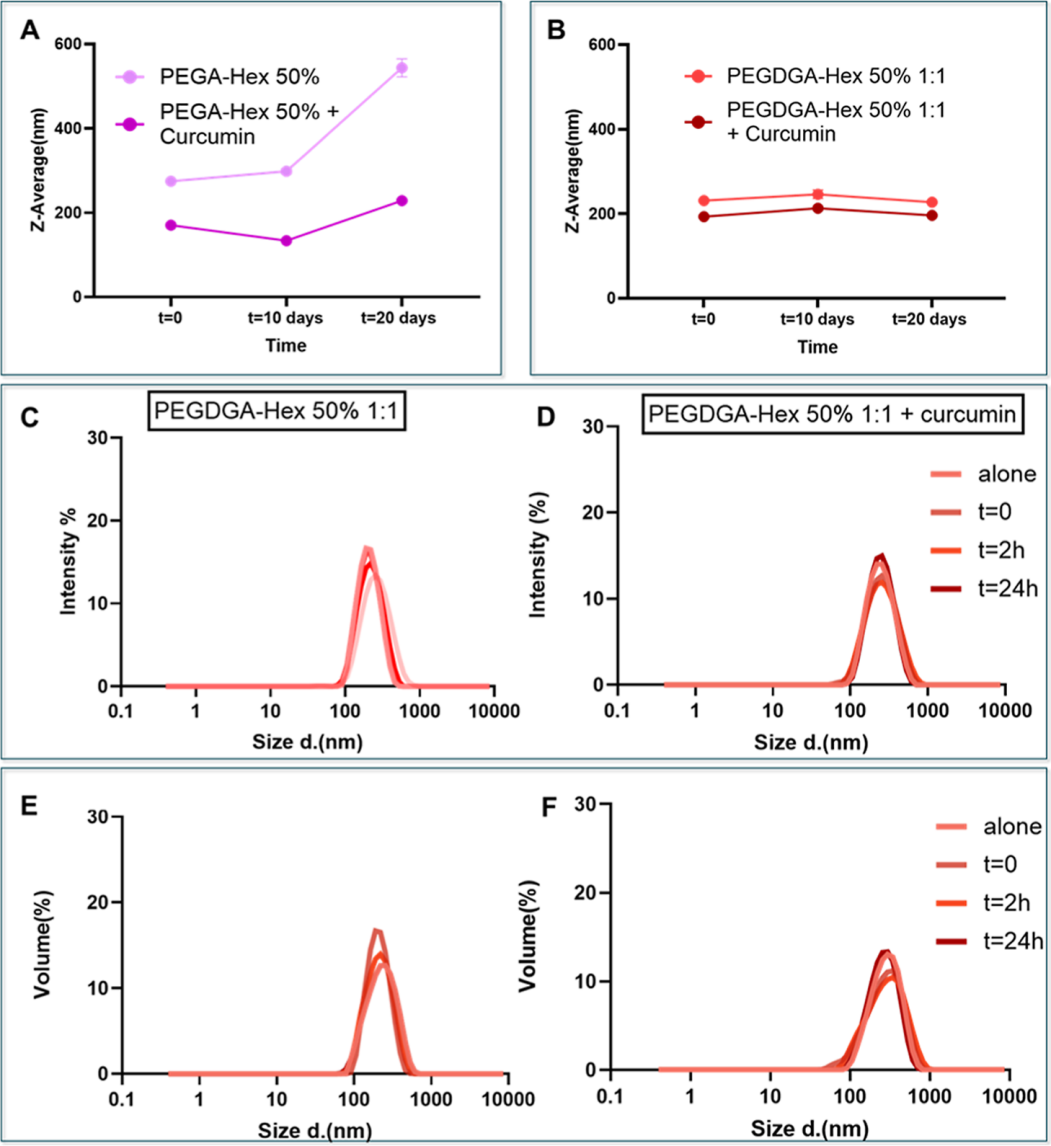
NP formation and stability results. (I) DLS size measurements showing NP stability over time up to 20 days for (A) PEGA-Hex 50% and (B) PEGDGA-Hex 50% 1:1 as free polymeric systems or curcumin-encapsulated systems. (II) DLS plots for PEGDGA-Hex 50% 1:1 for stability studies under biologically relevant conditions in BSA for (C) intensity plot of PEGDGA-Hex 50% and free polymeric systems, and (D) intensity plot of curcumin-encapsulated PEGDGA-Hex 50% 1:1. (E) Volume plot of PEGDGA-Hex 50% and free polymeric systems, and (F) volume plot of curcumin-encapsulated PEGDGA-Hex 50% 1:1. All plots are the average of three different runs.

Furthermore, both PEGA-Hex and PEGA-Hept were found to be more effective than PEGA-Quin at inhibiting the growth of *C. albicans* and *C. neoformans*. 50% and PEGDGA-Hex 50% 1:1 NPs exhibited enhanced stability when loaded with curcumin, as evidenced by reduced size fluctuations compared to their drug-free counterparts (Figure 4A,B). As previously discussed, this stabilizing effect can be attributed to the hydrophobic interactions between curcumin and the core-forming hydrophobic segments of the polymers, which likely contribute to a more compact and cohesive NP structure, thereby minimizing water penetration and aggregation over time.

### NP Stability in Biological Conditions

To assess the stability and stealth-like behavior of the NPs under biologically relevant conditions, BSA was employed as a model protein, due to its structural and functional similarity to human serum albumin present in the bloodstream.^93,94^ The stability of the NPs in the presence of BSA was evaluated by the DLS. Compared to the Control, PEGA-Hex 50% and PEGDGA-Hex 50% 1:1 exhibited higher NP stability under biologically relevant conditions (Figures 4C and S9). In both the absence and presence of curcumin, PEGDGA-Hex 50% 1:1 maintained a consistent size distribution across all time points (0, 2, and 24 h), indicating minimal aggregation. In contrast, PEGA-Hex 50% showed a noticeable broadening and shift in particle size over time, particularly at 24 h, suggesting reduced colloidal stability (Figures 4D and S10). The improved stability of PEGDGA-Hex is shown. 50% 1:1 may be attributed to the presence of diglycerol units, as previously stated, introducing additional hydrogen bonding interactions. These structural features may contribute to a more robust NP architecture, reducing the level of aggregation and improving dispersion stability over time.

The minor differences observed between intensity and volume distributions across time points further support colloidal stability; the volume plots show nearly identical size distributions, confirming that the bulk NP population remains unchanged, while the slight broadening in intensity plots reflects the sensitivity of this weighting to trace populations of larger species, consistent with minimal and nonprogressive protein corona formation.

To further confirm these observations, volume distributions were also recorded alongside intensity plots, as volume weighting provides a more representative measure of the bulk NP population by reducing the disproportionate contribution of trace larger species inherent to intensity-based measurements, as shown in Figure 4E,F.

### NP Qualitative Degradation Assay

To evaluate the susceptibility of the formulated NPs to enzymatic degradation, DLS was employed as a rapid screening method to monitor changes in the particle size following exposure to a lipase system (Figure 5A). This assay was designed to qualitatively assess whether the synthesized polymers are prone to enzymatic breakdown, rather than to quantify the extent of degradation. This approach aligns with previous literature, which suggests that DLS can serve as an effective preliminary tool for detecting polymer destabilization in the presence of lipolytic enzymes.^95^

**Figure 5:**
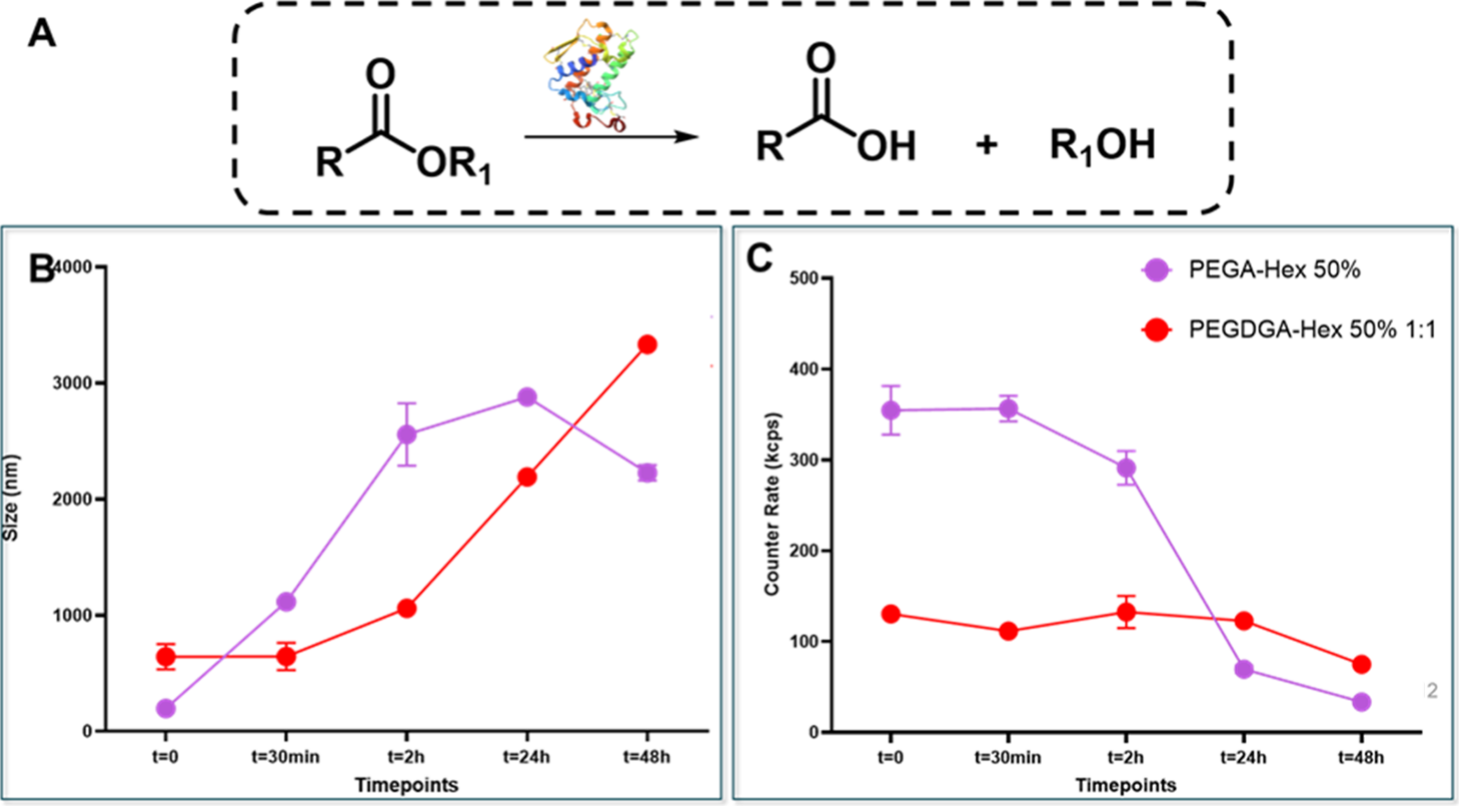
Enzymatic degradation results. (A) Schematic illustration of the enzymatic degradation using lipase. (B) Size and (C) count rate evolution during the enzymatic NP degradation studies.

Although PEG is widely used in biomedical applications due to its biocompatibility and stealth properties, it is generally considered nonbiodegradable.^50,96^ However, the incorporation of degradable polyester segments into PEG-based copolymers has been explored as a strategy to impart enzymatic degradability.^61,97^ To investigate this, the PEG-based polyesters PEGA-Hex were investigated. PEGA-Hex 50% and PEGDGA-Hex 50% 1:1 were evaluated for their susceptibility to enzymatic degradation using DLS.

The first parameter evaluated was the count rate, which reflects the scattering intensity normalized by the attenuation factor. According to the literature, ongoing enzymatic degradation typically results in a progressive decrease in count rate over time,^98–101^ a trend that was, in part, also observed in this study (Figure 5). A substantial drop in the count rate is indicative of NP degradation and sedimentation.^102^ PEGA-Hex 50% showed a sharp decline in count rate from ∼350 kcps at 0 h to below 50 kcps at 48 h. PEGDGA-Hex 50% 1:1 showed a plateauing behavior with a slight decline only after 48 h (Figure 5C).

The second parameter assessed was the particle size evolution. Both PEGA-Hex 50% and PEGDGA-Hex 50% 1:1 exhibited a marked increase in size over time, consistent with NP swelling and subsequent destabilization. Both polymers showed pronounced size increase, from approximately 300 nm at 0 h to over 2000 nm by 48 h, suggesting rapid enzymatic interaction and degradation (Figure 5B). The dramatic size increase is indicative of NP swelling, aggregation, and eventual destabilization as enzymatic cleavage of ester bonds disrupts the polymer network; however, these values are taken only as visual, qualitative cues of the degradation process. In fact, fluctuations and unreliability of the actual size, well beyond DLS resolution, cannot be used for quantitative analysis and full descriptors of the actual degradation steps.

These findings confirm that both PEG-based polyesters are susceptible to enzymatic degradation. The inclusion of diglycerol units in PEGDGA-Hex may promote hydrogen bonding between hydrophilic groups (e.g., PEG and glycerol/diglycerol), potentially leading to a more compact and sterically hindered structure. This may reduce enzyme accessibility and contribute to the slower degradation profile observed compared with that of PEGA-Hex. Nonetheless, both systems demonstrate clear biodegradability, distinguishing them from the nondegradable PEG control.

### Biocompatibility In Vitro and In Vivo

The cytocompatibility of the previously reported best-performing NP system, PEGA-Hex, was evaluated. PEGA-Hex 50% and the newly optimized tetrapolymer, PEGDGA-Hex 50% 1:1, was assessed. Caco-2 human intestinal epithelial cells were exposed for 24 h to three polymer concentrations (0.25, 0.5, and 2.5 mg mL^–1^) (Figure 6). Cell viability was assessed by measuring metabolic activity. Since the systems are amphiphilic and assemble in nanosystems, we also evaluated the plasma membrane integrity through extracellular lactate dehydrogenase (LDH) release (Figure 6B). Across all tested concentrations, neither polymeric system exhibited any cytotoxic effects, as evidenced by the absence of significant change in metabolic activity or LDH release relative to the vehicle control (DMEM).

**Figure 6:**
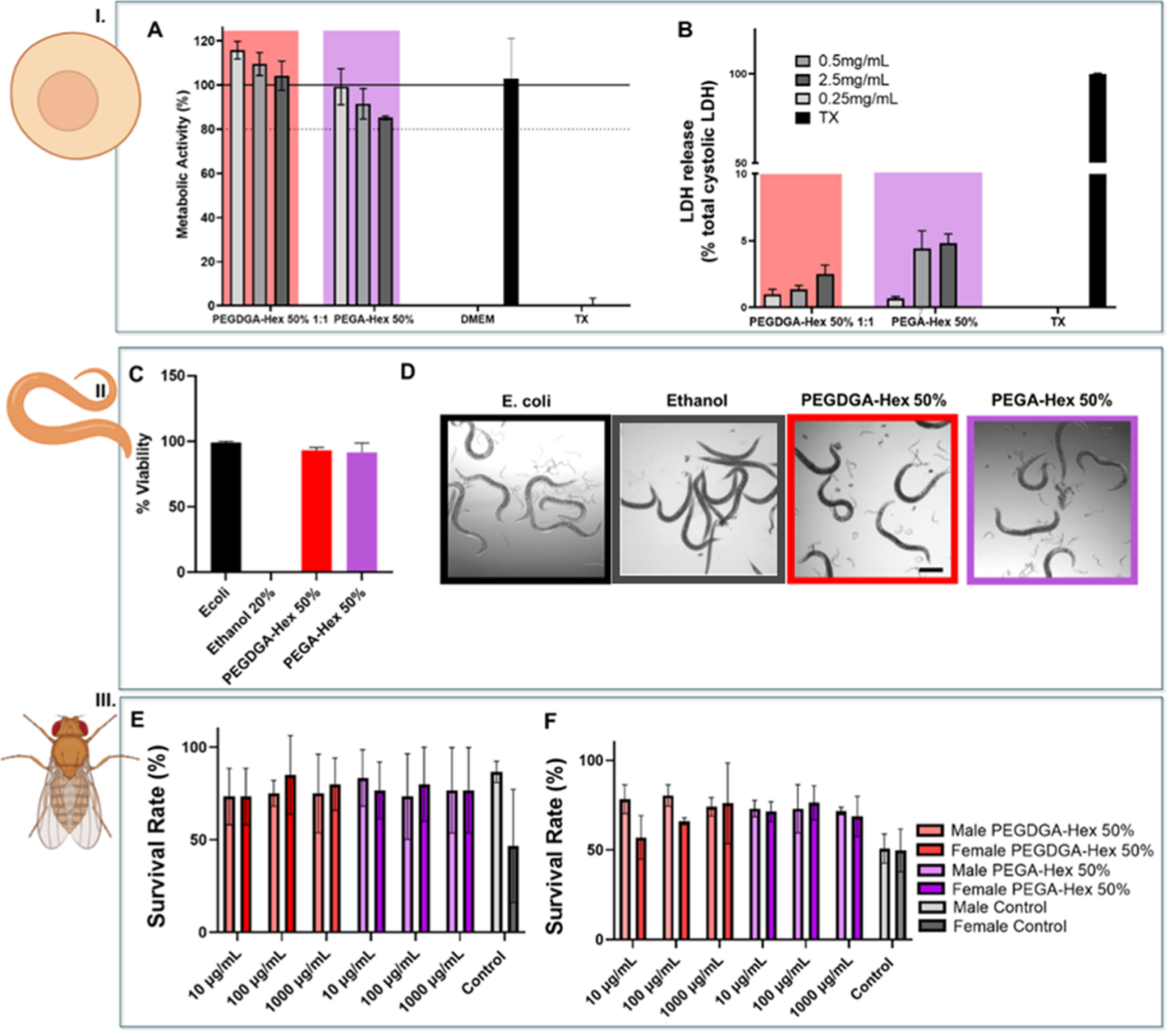
Cytotoxicity of tested polymeric systems. (I) In vitro assessment in human intestinal Caco-2 cells. (A) Metabolic activity using PrestoBlue assay and (B) LDH release assay. Cells were exposed for 24 h to varying concentrations of polymer NPs and DMEM culture media as vehicle control and 1% (v/v) Triton X-100 (TX) as cell death-inducing control. Data are presented as mean ± SD. Statistical analysis using a two-way ANOVA test determined no significant difference between test systems and DMEM control in both assays (*P* > 0.05). (II) In vivo assessment using *C. elegans*. (C,D) Viability of *C. elegans* was assessed after 24 h exposure to polymeric nanoparticles (0.5 mg mL^–1^), evaluated as a function of motility. All treatments were tested in triplicate with 20–30 worms per well. No statistically significant differences between the NP treatments and viable control (*E. coli*, *P* > 0.05), whereas ethanol exposure led to a significant loss of viability (*P* < 0.001). (III) In vivo assessment using *Drosophila melanogaster*. (E) Survival rate of parents after a two-week exposure to the polymeric systems. (F) Survival rate of F1 after a six-week exposure to the same polymeric systems.

To further emphasize the nontoxicity of these materials, the same experiments were conducted using breast cancer cell lines, MCF-7 and MDA-MB-231, with varying degrees of aggressiveness and phenotypic characteristics. These complementary assays confirmed the biocompatibility of both polymer systems across diverse cellular models, reinforcing their potential for safe biomedical applications (Figure S11).

Regarding the in vivo biocompatibility assessment, the first evaluation occurred using young adult *C. elegans* following 24 h incubation, using *E. coli* OP50 and ethanol (20% v/v) as viable and nonviable controls, respectively. Nematodes exposed to *E. coli* OP50 exhibited growth consistent with reported morphology,^103,104^ active locomotion, and progeny production, confirming viability.^69,71^ In contrast, ethanol-treated worms were immobile, unresponsive to stimulation, and produced no progeny, consistent with acute lethality. Nematodes were incubated with both PEGA-Hex 50% and PEGDA-Hex 50% 1:1 remained viable after 24 h with no significant differences when compared to the *E. coli* control, *P* > 0.05 (Figure 6C,D). Worms in both polymer treatments also produced progeny, indicating preserved reproductive capacity and the absence of severe physiological stress (Figure S12). The maintenance of viability, locomotion, and progeny production demonstrates that both PEGA-Hex and PEGDA-Hex 50% 1:1 are biocompatible with adult *C. elegans* over 24 h. These findings support their suitability for further biological applications and underscore the utility of *C. elegans* as a sensitive whole-organism model for early stage biocompatibility assessment of polymeric materials.

These findings were further corroborated in *D. melanogaster*, providing additional in vivo confirmation of the overall biocompatibility profile observed in both the in vitro and *C. elegans* models. The parental (P) generation exhibited no significant differences in survival following the two-week exposure period (Figure 6E), further demonstrating that the polymeric systems did not induce acute or short-term toxicity in adult *D. melanogaster*. Similarly, the survival rate of the F1 generation was maintained over the six-week observation period, with no significant reduction compared to the control group (Figures 6F and S13). Although a gradual decline in survival was observed over time, this trend was consistent across all groups and followed the expected pattern of age-related mortality. Importantly, no dose-dependent or polymer-specific adverse effects were detected. Furthermore, the climbing assay (Figure S13B) demonstrated that locomotor performance in all polymer-exposed groups closely mirrored that of the control throughout the study, indicating that exposure to the polymeric systems did not impair neuromuscular function. Collectively, these data further substantiate the nontoxic nature of PEGA-Hex 50% and PEGDA-Hex 50% 1:1 and confirm their in vivo safety across multiple biological models.

## Conclusions

This study extended the established enzymatic route for producing PEGylated polyesters by incorporating bioderived polyols, such as glycerol and diglycerol, previously examined within the same catalytic framework. We demonstrated the successful synthesis of minimally PEGylated tetrapolymers and highlighted the potential of this approach for supporting the development of more sustainable polyester systems. These fine-tuned self-assemblies of these tetrapolymers into NPs from SD using film rehydration, without the addition of secondary organic solvent and surfactants. The efficient encapsulation of curcumin, a model hydrophobic drug, was demonstrated using SD, showcasing these polymers as innovative and environmentally friendly hybrid, nanodrug delivery systems.

The development of these new tetrapolymeric SDs was compared to the best-performing PEGylated polymer identified in a previous study. The apparent solubility of curcumin in the best-performing tetrapolymeric nanoformulation was comparable to that of the best-performing PEGylated control system, demonstrating that by reducing the PEG content of polymer excipients by 50%, we did not compromise the encapsulation efficiency. Furthermore, the stability of both loaded and unloaded tetrapolymeric NPs was shown to be higher than that of the control with a higher amount of PEG. Finally, the tetrapolymers were shown to be nontoxic in in vitro testing and in in vivo invertebrate models, *C. elegans* and *D. melanogaster*, which adhere to the principles of replacement, reduction, and refinement (3Rs). This study demonstrated that over-reliance on PEG-based polymers can be avoided, and by minimizing the amount of PEG used in copolymers, the benefits of PEG-based systems can still be exploited, offering a promising strategy for the encapsulation of hydrophobic drugs through SDs.

## Supplementary Material



## Notes

^1^H NMR spectra of the new tetrapolymers PEGGA–Hex (50% 1:1) and PEGDGA–Hex (50% 1:1), as well as samples with different diglycerol ratios, ^13^C NMR and 2D NMR spectra of the tetrapolymer PEGDGA–Hex (50% 1:1), GPC traces of the tetrapolymers and terpolymer analyzed in this work, DSC trace of PEGDGA–Hex (50% 1:1), DLS analysis of empty and curcumin-loaded nanoparticles, reporting Z-average size, PDI, and attenuator values, SAXS analysis of empty and curcumin-loaded nanoparticles, DLS stability measurements of nanoparticles under biologically relevant conditions, cytotoxicity profiles of nanoparticles in MCF-7 (human breast cancer) and MDA-MB-231 (triple-negative breast cancer) cell lines, representative microscopy images of *C. elegans* 24 h after exposure to polymer nanoparticles, and survival rates of female and male flies over 6 weeks of nanoparticle exposure (PDF)

## References

[1] Mitragotri S., Burke P. A., Langer R. (2014). Overcoming the Challenges in Administering
Biopharmaceuticals: Formulation and Delivery Strategies. Nat. Rev. Drug Discov..

[2] Ristroph K. D. (2024). Drugs Need
to Be Formulated with Scale-up in Mind. J. Controlled
Release.

[3] Xie B., Liu Y., Li X., Yang P., He W. (2024). Solubilization Techniques
Used for Poorly Water-Soluble Drugs. Acta Pharm.
Sin. B.

[5] Kumari L., Choudhari Y., Patel P., Gupta G. D., Singh D., Rosenholm J. M., Bansal K. K., Kurmi B. D. (2023). Advancement in Solubilization
Approaches: A Step towards Bioavailability Enhancement of Poorly Soluble
Drugs. Life.

[6] Kalepu S., Nekkanti V. (2015). Insoluble Drug Delivery Strategies: Review of Recent
Advances and Business Prospects. Acta Pharm.
Sin. B.

[7] Mahmood T., Sarfraz R. M., Ismail A., Ali M., Khan A. R. (2023). Pharmaceutical
Methods for Enhancing the Dissolution of Poorly Water-Soluble Drugs. Assay Drug Dev. Technol..

[8] Hiew T. N., Taylor L. S. (2022). Combining Drug Salt Formation with Amorphous Solid
Dispersions – a Double Edged Sword. J.
Controlled Release.

[9] Budiman A., Handini A. L., Muslimah M. N., Nurani N. V., Laelasari E., Kurniawansyah I. S., Aulifa D. L. (2023). Amorphous Solid Dispersion as Drug
Delivery Vehicles in Cancer. Polymers.

[10] Iyer R., Petrovska Jovanovska V., Berginc K., Jaklič M., Fabiani F., Harlacher C., Huzjak T., Sanchez-Felix M. V. (2021). Amorphous
Solid Dispersions (ASDs): The Influence of Material Properties, Manufacturing
Processes and Analytical Technologies in Drug Product Development. Pharmaceutics.

[11] Hussein Y. H. A., Youssry M. (2018). Polymeric Micelles
of Biodegradable Diblock Copolymers:
Enhanced Encapsulation of Hydrophobic Drugs. Materials.

[12] Kim S. C., Kim D. W., Shim Y. H., Bang J. S., Oh H. S., Kim S. W., Seo M. H. (2001). In Vivo
Evaluation of Polymeric Micellar
Paclitaxel Formulation: Toxicity and Efficacy. J. Controlled Release.

[13] Malkawi R., Malkawi W. I., Al-Mahmoud Y., Tawalbeh J. (2022). Current Trends on Solid
Dispersions: Past, Present, and Future. Adv.
Pharmacol. Pharm. Sci..

[14] Sareen S., Mathew G., Joseph L. (2012). Improvement in Solubility of Poor
Water-Soluble Drugs by Solid Dispersion. Int.
J. Pharm. Investig..

[15] Martínez
Rivas C. J., Tarhini M., Badri W., Miladi K., Greige-Gerges H., Nazari Q. A., Galindo Rodríguez S. A., Román R. Á., Fessi H., Elaissari A. (2017). Nanoprecipitation
Process: From Encapsulation to Drug Delivery. Int. J. Pharm..

[16] Yarlagadda D. L., Sai Krishna Anand V., Nair A. R., Navya Sree K. S., Dengale S. J., Bhat K. (2021). Considerations for the Selection
of Co-Formers in the Preparation of Co-Amorphous Formulations. Int. J. Pharm..

[17] Bhujbal S. V., Mitra B., Jain U., Gong Y., Agrawal A., Karki S., Taylor L. S., Kumar S., Tony Zhou Q. (2021). Pharmaceutical
Amorphous Solid Dispersion: A Review of Manufacturing Strategies. Acta Pharm. Sin. B.

[18] Pandi P., Bulusu R., Kommineni N., Khan W., Singh M. (2020). Amorphous
Solid Dispersions: An Update for Preparation, Characterization, Mechanism
on Bioavailability, Stability, Regulatory Considerations and Marketed
Products. Int. J. Pharm..

[19] Tran P., Pyo Y.-C., Kim D.-H., Lee S.-E., Kim J.-K., Park J.-S. (2019). Overview of the Manufacturing Methods of Solid Dispersion
Technology for Improving the Solubility of Poorly Water-Soluble Drugs
and Application to Anticancer Drugs. Pharmaceutics.

[20] Ladan
Akbarpour Nikghalb Gaurav Singh G. S., Kahkeshan K. F. (2012). Solid Dispersion:
Methods and Polymers to Increase the Solubility of Poorly Soluble
Drugs. J. Appl. Pharmaceut. Sci..

[21] Sharma A., Jain C. P. (2010). Preparation and Characterization of Solid Dispersions
of Carvedilol with PVP K30. Res. Pharm. Sci..

[22] Patil H., Vemula S. K., Narala S., Lakkala P., Munnangi S. R., Narala N., Jara M. O., Williams R. O., Terefe H., Repka M. A. (2024). Hot-Melt Extrusion:
From Theory to Application in Pharmaceutical
Formulation–Where Are We Now?. AAPS PharmSciTech.

[23] Singh A., Van den Mooter G. (2016). Spray Drying
Formulation of Amorphous Solid Dispersions. Adv. Drug Delivery Rev..

[24] Paudel A., Worku Z. A., Meeus J., Guns S., Van den
Mooter G. (2013). Manufacturing of Solid Dispersions of Poorly Water Soluble Drugs
by Spray Drying: Formulation and Process Considerations. Int. J. Pharm..

[25] Mann A. K. P., Schenck L., Koynov A., Rumondor A. C. F., Jin X., Marota M., Dalton C. (2018). Producing Amorphous
Solid Dispersions
via Co-Precipitation and Spray Drying: Impact to Physicochemical and
Biopharmaceutical Properties. J. Pharm. Sci..

[26] Almeida H., Ferreira B., Fernandes-Lopes C., Araújo F., Bonifácio M. J., Vasconcelos T., Sarmento B. (2024). Third-Generation Solid
Dispersion Through Lyophilization Enhanced Oral Bioavailability of
Resveratrol. ACS Pharmacol. Transl. Sci..

[27] Almeida H., Teixeira N., Sarmento B., Vasconcelos T. (2024). Freeze-Drying
Cycle Optimization of an Amorphous Solid Dispersion of Resveratrol. Eur. J. Pharm. Sci..

[28] Strotman N. A., Schenck L. (2022). Coprecipitated Amorphous
Dispersions as Drug Substance:
Opportunities and Challenges. Org. Process Res.
Dev..

[29] Cao J., Zhang S., Hao Y., Fan K., Wang L., Zhao X., He X. (2023). Amorphous Solid Dispersion
Preparation
via Coprecipitation Improves the Dissolution, Oral Bioavailability,
and Intestinal Health Enhancement Properties of Magnolol. Poult. Sci..

[30] Hu Z., Xu P., Ashour E. A., Repka M. A. (2022). Prediction and Construction of Drug-Polymer
Binary System Thermodynamic Phase Diagram in Amorphous Solid Dispersions
(ASDs). AAPS PharmSciTech.

[31] Tian H., Zhang T., Qin S., Huang Z., Zhou L., Shi J., Nice E. C., Xie N., Huang C., Shen Z. (2022). Enhancing
the Therapeutic Efficacy of Nanoparticles for Cancer Treatment Using
Versatile Targeted Strategies. J. Hematol. Oncol..

[32] Ricarte R. G., Van Zee N. J., Li Z., Johnson L. M., Lodge T. P., Hillmyer M. A. (2019). Recent Advances
in Understanding the Micro- and Nanoscale
Phenomena of Amorphous Solid Dispersions. Mol.
Pharmaceutics.

[33] Chan, J. M. ; Valencia, P. M. Polymeric Nanoparticles for Drug Delivery. In Cancer Nanotechnology: Methods and Protocols; Grobmyer, S. R. , Moudgil, B. M. , Eds.; Humana Press: Totowa, NJ, 2010; pp 163–175. 10.1007/978-1-60761-609-2_11 20217595

[34] Panchal S. S., Vasava D. V. (2020). Biodegradable Polymeric Materials: Synthetic Approach. ACS Omega.

[35] Sung Y. K., Kim S. W. (2020). Recent Advances in Polymeric Drug
Delivery Systems. Biomater. Res..

[36] Floyd T. G., Gurnani P., Rho J. Y. (2025). Characterisation
of Polymeric Nanoparticles
for Drug Delivery. Nanoscale.

[37] Kumari A., Yadav S. K., Yadav S. C. (2010). Biodegradable Polymeric Nanoparticles
Based Drug Delivery Systems. Colloids Surf.,
B.

[38] Bilati U., Allémann E., Doelker E. (2005). Development of a Nanoprecipitation
Method Intended for the Entrapment of Hydrophilic Drugs into Nanoparticles. Eur. J. Pharm. Sci..

[39] Wilson R. J., Li Y., Yang G., Zhao C.-X. (2022). Nanoemulsions
for Drug Delivery. Particuology.

[40] Souto E. B., Cano A., Martins-Gomes C., Coutinho T. E., Zielińska A., Silva A. M. (2022). Microemulsions and
Nanoemulsions in Skin Drug Delivery. Bioengineering.

[41] D̵oković J. B., Savić S. M., Mitrović J. R., Nikolic I., Marković B. D., Randjelović D. V., Antic-Stankovic J., Božić D., Cekić N. D., Stevanović V., Batinić B., Arand̵elović J., Savić M. M., Savić S. D. (2021). Curcumin Loaded PEGylated Nanoemulsions Designed for
Maintained Antioxidant Effects and Improved Bioavailability: A Pilot
Study on Rats. Int. J. Mol. Sci..

[42] Calderó G., García-Celma M. J., Solans C. (2011). Formation of Polymeric
Nano-Emulsions by a Low-Energy Method and Their Use for Nanoparticle
Preparation. J. Colloid Interface Sci..

[43] Stepanova D., Pigareva V., Berkovich A., Bolshakova A., Spiridonov V., Grozdova I., Sybachin A. (2022). Ultrasonic
Film Rehydration
Synthesis of Mixed Polylactide Micelles for Enzyme-Resistant Drug
Delivery Nanovehicles. Polymers.

[44] Baghel S., Cathcart H., O’Reilly N. (2016). Polymeric Amorphous Solid Dispersions:
A Review of Amorphization, Crystallization, Stabilization, Solid-State
Characterization, and Aqueous Solubilization of Biopharmaceutical
Classification System Class II Drugs. J. Pharm.
Sci..

[45] Van
Den Mooter G. (2012). The Use of Amorphous Solid Dispersions: A Formulation
Strategy to Overcome Poor Solubility and Dissolution Rate. Drug Discovery Today:Technol..

[46] Bayraktutan H., Symonds P., Brentville V. A., Moloney C., Galley C., Bennett C. L., Mata A., Durrant L., Alexander C., Gurnani P. (2024). Sparsely PEGylated
Poly­(Beta-Amino Ester) Polyplexes
Enhance Antigen Specific T-Cell Response of a Bivalent SARS-CoV-2
DNA Vaccine. Biomaterials.

[47] De
Leo V., Maurelli A., Giotta L., Catucci L. (2022). Liposomes Containing
Nanoparticles: Preparation and Applications. Colloids Surf., B.

[48] Kawakami K. (2025). Roles of Supersaturation
and Liquid–Liquid Phase Separation for Enhanced Oral Absorption
of Poorly Soluble Drugs from Amorphous Solid Dispersions. Pharmaceutics.

[49] Ibrahim M., Ramadan E., Elsadek N. E., Emam S. E., Shimizu T., Ando H., Ishima Y., Elgarhy O. H., Sarhan H. A., Hussein A. K., Ishida T. (2022). Polyethylene
Glycol (PEG): The Nature,
Immunogenicity, and Role in the Hypersensitivity of PEGylated Products. J. Controlled Release.

[50] Hoang
Thi T. T., Pilkington E. H., Nguyen D. H., Lee J. S., Park K. D., Truong N. P. (2020). The Importance of Poly­(Ethylene Glycol)
Alternatives for Overcoming PEG Immunogenicity in Drug Delivery and
Bioconjugation. Polymers.

[51] Turecek P. L., Bossard M. J., Schoetens F., Ivens I. A. (2016). PEGylation of Biopharmaceuticals:
A Review of Chemistry and Nonclinical Safety Information of Approved
Drugs. J. Pharm. Sci..

[52] Gao Y., Joshi M., Zhao Z., Mitragotri S. (2024). PEGylated
Therapeutics in the Clinic. Bioeng. Transl.
Med..

[53] Liu Y., Liu C., An D., Deng S., Liu G. (2025). Engineered Nanomaterials
for Overcoming Multifaceted Gastrointestinal Barriers: Toward Precision
Oral Delivery of Therapeutics. Pharmacol. Res..

[54] Harris J. M., Chess R. B. (2003). Effect of Pegylation on Pharmaceuticals. Nat. Rev. Drug Discovery.

[55] Ou X., Li S., Chen Y., Rong H., Li A., Lu M. (2022). Polymorphism
in Griseofulvin: New Story between an Old Drug and Polyethylene Glycol. Cryst. Growth Des..

[56] Helsinta N., Halim A., Octavia M. D., Rivai H. (2021). Review: Solid Dispersion
of Fenofibrate Using Poly Ethylene Glycol 6000. Int. J. Med. Pharmaceut. Sci..

[57] Ling A. (2013). Review of
Currently Available Fenofibrate and Fenofibric Acid Formulations. Cardiol. Res..

[58] Kamaly N., Yameen B., Wu J., Farokhzad O. C. (2016). Degradable
Controlled-Release Polymers and Polymeric Nanoparticles: Mechanisms
of Controlling Drug Release. Chem. Rev..

[59] Tejwani R. W., Joshi H. N., Varia S. A., Serajuddin A. T. M. (2000). Study
of Phase Behavior of Poly­(Ethylene Glycol)–Polysorbate 80 and
Poly­(Ethylene Glycol)–Polysorbate 80–Water Mixtures. J. Pharm. Sci..

[60] Suk J. S., Xu Q., Kim N., Hanes J., Ensign L. M. (2016). PEGylation as a
Strategy for Improving Nanoparticle-Based Drug and Gene Delivery. Adv. Drug Deliv. Rev..

[61] Axioti E., Dixon E. G., Jepras T., Tin He F., Hartman P. J. V., Hopkins B., Di Bari V., Suksiriworapong J., Cuzzucoli Crucitti V., Galantini L., Francolini I., Cavanagh R. J., Taresco V. (2025). Enzymatic Synthesis of Functional
PEGylated Adipate Copolymers. ChemPlusChem.

[62] Lin D., Huang Y. (2010). A Thermal Analysis
Method to Predict the Complete Phase Diagram of
Drug–Polymer Solid Dispersions. Int.
J. Pharm..

[63] Srividya B., Ghosh A. (2025). Mechanistic Insights
into Amorphous Solid Dispersions: Bridging Theory
and Practice in Drug Delivery. Pharm. Res..

[64] Knop K., Hoogenboom R., Fischer D., Schubert U. S. (2010). Poly­(Ethylene Glycol)
in Drug Delivery: Pros and Cons as Well as Potential Alternatives. Angew. Chem., Int. Ed..

[65] Shah J. C., Chen J. R., Chow D. (1995). Preformulation Study of Etoposide:
II. Increased Solubility and Dissolution Rate by Solid-Solid Dispersions. Int. J. Pharm..

[66] Khan A., Sharma S., Kumar A., Watterson A., Kumar J., Parmar V. (2014). Novozym 435-Catalyzed Syntheses of
Polyesters and Polyamides of Medicinal and Industrial Relevance. ChemSusChem.

[67] Jacob P. L., Ruiz Cantu L. A., Pearce A. K., He Y., Lentz J. C., Moore J. C., Machado F., Rivers G., Apebende E., Fernandez M. R., Francolini I., Wildman R., Howdle S. M., Taresco V. (2021). Poly (Glycerol
Adipate) (PGA) Backbone Modifications
with a Library of Functional Diols: Chemical and Physical Effects. Polymer.

[68] Taresco V., Creasey R., Kennon J., Mantovani G., Alexander C., Burley J., Garnett M. (2016). Variation
in Structure
and Properties of Poly­(Glycerol Adipate) via Control of Chain Branching
during Enzymatic Synthesis. Polymer.

[69] Axioti E., Dixon E. G., Reynolds-Green M., Alexander E. C. H., Brugnoli B., Keddie D. J., Couturaud B., Suksiriworapong J., Swainson S. M. E., Francolini I., Howdle S. M., Jacob P. L., Cavanagh R. J., Chauhan V. M., Taresco V. (2024). Glycerol- and Diglycerol-Based Polyesters: Evaluation
of Backbone Alterations upon Nano-Formulation Performance. Colloids Surf., B.

[70] D’Anna P., Jacob P. L., Axioti E., Alvey K. E., Berfi N. A., Pearce J. E., Cavanagh R. J., Chauhan V. M., Taresco V., Conte C. (2024). Glycerol-Based Copolyesters
as Polymeric Nanocarriers for Drug Delivery. J. Polym. Sci..

[71] Jacob P., Brugnoli B., Del Giudice A., Phan H., Chauhan V., Beckett L., Gillis R., Moloney C., Cavanagh R., Krumins E., Reynolds-Green M., Lentz J. C., Conte C., Cuzzucoli Crucitti V., Couturaud B., Galantini L., Francolini I., Howdle S., Taresco V. (2023). Poly (Diglycerol Adipate)
Variants as Enhanced Nanocarrier Replacements in Drug Delivery Applications. J. Colloid Interface Sci..

[72] Cristiano C., Cavanagh R. J., Cuzzucoli
Crucitti V., Moloney C., Axioti E., Dixon E., Jacob P. L., Schiano M. E., Cuozzo M., Liguori F. M., Rolando B., Russo R., Taresco V., Sodano F., Rimoli M. G. (2024). Multiple Drug-Delivery Strategies
to Enhance the Pharmacological and Toxicological Properties of Mefenamic
Acid. Biomed. Pharmacother..

[73] Saller K. M., Pernusch D. C., Schwarzinger C. (2023). MALINTO: A New MALDI Interpretation
Tool for Enhanced Peak Assignment and Semiquantitative Studies of
Complex Synthetic Polymers. J. Am. Soc. Mass
Spectrom..

[74] Smith A. J., Alcock S. G., Davidson L. S., Emmins J. H., Hiller
Bardsley J. C., Holloway P., Malfois M., Marshall A. R., Pizzey C. L., Rogers S. E., Shebanova O., Snow T., Sutter J. P., Williams E. P., Terrill N. J. (2021). I22: SAXS/WAXS
Beamline at Diamond Light Source - an Overview of 10 Years Operation. J. Synchrotron Radiat..

[75] Pauw B. R., Smith A. J., Snow T., Terrill N. J., Thünemann A. F. (2017). The Modular
Small-Angle X-Ray Scattering Data Correction Sequence. J. Appl. Crystallogr..

[76] Filik J., Ashton A. W., Chang P. C. Y., Chater P. A., Day S. J., Drakopoulos M., Gerring M. W., Hart M. L., Magdysyuk O. V., Michalik S., Smith A., Tang C. C., Terrill N. J., Wharmby M. T., Wilhelm H. (2017). Processing Two-Dimensional
X-Ray
Diffraction and Small-Angle Scattering Data in DAWN 2. J. Appl. Crystallogr..

[77] Chauhan V. M., Orsi G., Brown A., Pritchard D. I., Aylott J. W. (2013). Mapping the Pharyngeal and Intestinal PH of Caenorhabditis
Elegans and Real-Time Luminal PH Oscillations Using Extended Dynamic
Range PH-Sensitive Nanosensors. ACS Nano.

[78] Slankster E., Kollala S., Baria D., Dailey-Krempel B., Jain R., Odell S. R., Mathew D. (2020). Mechanism
Underlying
Starvation-Dependent Modulation of Olfactory Behavior in Drosophila
Larva. Sci. Rep..

[79] Chatterjee A., Roman G., Hardin P. E. (2009). Go Contributes to
Olfactory Reception
in Drosophila Melanogaster. BMC Physiol..

[80] Bolukbasi E., Woodling N. S., Ivanov D. K., Adcott J., Foley A., Rajasingam A., Gittings L. M., Aleyakpo B., Niccoli T., Thornton J. M., Partridge L. (2021). Cell Type-Specific
Modulation of
Healthspan by Forkhead Family Transcription Factors in the Nervous
System. Proc. Natl. Acad. Sci. U. S. A..

[81] Mahadik N., Paruchuri S. N., Arif R., Coutts A. S., Barron G. A., Kong
Thoo Lin P., Chatterjee S., Thompson C. J. (2025). Evaluation of the
Toxicity and Efficacy of a Multi-Target Polymer-Drug Nano-Polyplex
in SH-SY5Y Cells and Drosophila Model of Tauopathy. Sci. Rep..

[82] Yang Y., Zhang J., Wu D., Xing Z., Zhou Y., Shi W., Li Q. (2014). Chemoenzymatic
Synthesis of Polymeric Materials Using
Lipases as Catalysts: A Review. Biotechnol.
Adv..

[83] Puri S., Kallinteri P., Higgins S., Hutcheon G. A., Garnett M. C. (2008). Drug Incorporation
and Release of Water Soluble Drugs from Novel Functionalised Poly­(Glycerol
Adipate) Nanoparticles. J. Controlled Release.

[84] Taresco V., Suksiriworapong J., Creasey R., Burley J. C., Mantovani G., Alexander C., Treacher K., Booth J., Garnett M. C. (2016). Properties
of Acyl Modified Poly­(Glycerol-Adipate) Comb-like Polymers and Their
Self-Assembly into Nanoparticles. J. Polym.
Sci. A Polym. Chem..

[85] Martinelli E., Galli G., Cwikel D., Marmur A. (2012). Wettability and Surface
Tension of Amphiphilic Polymer Films: Time-Dependent Measurements
of the Most Stable Contact Angle. Macromol.
Chem. Phys..

[86] Lee W., Loo C. Y., Bebawy M., Luk F., Mason R., Rohanizadeh R. (2013). Curcumin and
Its Derivatives: Their Application in
Neuropharmacology and Neuroscience in the 21st Century. Curr. Neuropharmacol..

[87] Annunziata F., Pinna C., Dallavalle S., Tamborini L., Pinto A. (2020). An Overview of Coumarin as a Versatile
and Readily Accessible Scaffold
with Broad-Ranging Biological Activities. Int.
J. Mol. Sci..

[88] Adiwidjaja J., McLachlan A. J., Boddy A. V. (2017). Curcumin as a Clinically-Promising
Anti-Cancer Agent: Pharmacokinetics and Drug Interactions. Expert Opin. Drug Metab. Toxicol..

[89] Sun J., Bi C., Chan H. M., Sun S., Zhang Q., Zheng Y. (2013). Curcumin-Loaded
Solid Lipid Nanoparticles Have Prolonged in Vitro Antitumour Activity,
Cellular Uptake and Improved in Vivo Bioavailability. Colloids Surf., B.

[90] Rizvi S. A. A., Saleh A. M. (2018). Applications of
Nanoparticle Systems in Drug Delivery
Technology. Saudi Pharm. J..

[91] Dolai J., Mandal K., Jana N. R. (2021). Nanoparticle
Size Effects in Biomedical
Applications. ACS Appl. Nano Mater..

[92] Zhu D., Yan H., Zhou Y., Nack L. M., Liu J., Parak W. J. (2023). Design
of Disintegrable Nanoassemblies to Release Multiple Small-Sized Nanoparticles. Adv. Drug Delivery Rev..

[93] Polat H., Eren M. C., Polat M. (2021). The Effect
of Protein BSA on the
Stability of Lipophilic Drug (Docetaxel)-Loaded Polymeric Micelles. Colloids Surf. A Physicochem. Eng. Asp..

[94] Oliveira C. L., Veiga F., Varela C., Roleira F., Tavares E., Silveira I., Ribeiro A. J. (2017). Characterization of Polymeric Nanoparticles
for Intravenous Delivery: Focus on Stability. Colloids Surf., B.

[95] Swainson S. M. E., Taresco V., Pearce A. K., Clapp L. H., Ager B., McAllister M., Bosquillon C., Garnett M. C. (2019). Exploring the Enzymatic
Degradation of Poly­(Glycerol Adipate). Eur.
J. Pharm. Biopharm..

[96] Xu L., Yang J., Xue B., Zhang C., Shi L., Wu C., Su Y., Jin X., Liu Y., Zhu X. (2017). Molecular
Insights for the Biological Interactions between Polyethylene Glycol
and Cells. Biomaterials.

[97] Manavitehrani I., Fathi A., Badr H., Daly S., Negahi Shirazi A., Dehghani F. (2016). Biomedical Applications
of Biodegradable Polyesters. Polymers.

[98] Thomsen T. B., Almdal K., Meyer A. S. (2023). Significance of
Poly­(Ethylene Terephthalate)
(PET) Substrate Crystallinity on Enzymatic Degradation. N. Biotechnol..

[99] Cai Z., Li M., Zhu Z., Wang X., Huang Y., Li T., Gong H., Yan M. (2023). Biological Degradation of Plastics
and Microplastics: A Recent Perspective on Associated Mechanisms and
Influencing Factors. Microorganisms.

[100] Ma L., Fan Z.-Y., Lian W.-Q., Wei X.-F., Bao R.-Y., Yang W. (2025). Nanoplastics and Microplastics
Released from an Enzyme-Embedded Biodegradable
Polyester during Hydrolysis. J. Hazard. Mater..

[101] Mohanan N., Montazer Z., Sharma P. K., Levin D. B. (2020). Microbial
and Enzymatic Degradation of Synthetic Plastics. Front. Microbiol..

[102] de Gracia Lux C., Vezeridis A. M., Lux J., Armstrong A. M., Sirsi S. R., Hoyt K., Mattrey R. F. (2017). Novel Method
for
the Formation of Monodisperse Superheated Perfluorocarbon Nanodroplets
as Activatable Ultrasound Contrast Agents. RSC
Adv..

[103] Corsi A. K., Wightman B., Chalfie M. (2015). A Transparent
Window
into Biology: A Primer on Caenorhabditis Elegans. WormBook.

[104] Hunt P. R. (2017). The C. Elegans Model in Toxicity Testing. J. Appl. Toxicol..

