# Supplementary material for: Sparingly PEGylated Adipate Copolymers via Enzymatic Synthesis as Nano-Carriers for Solid Dispersions
Source: Biomacromolecules. 2026 Jun 5;27(7):4675–90. doi: 10.1021/acs.biomac.6c00647 (PMC13370786; doi:10.1021/acs.biomac.6c00647)
Supplement: Supplementary file 1 [file bm6c00647_si_001.pdf]

Supporting Information

Sparingly PEGylated Adipate Copolymers *via* Enzymatic Synthesis as Nano-Carriers for Solid Dispersions

Eleni Axioti [a], Nana A. Berfi [b], Philippa L. Jacob [a], Klara M. Saller [c], Georgia L. Maitland [d,e], Anisha Patel [d,e], Sri Nithya Paruchuri [f], Paul D. Topham [d,e], Matthew J. Derry [d,e], Shreyasi Chatterjee [f], Benoit Couturaud [g], Luciano Galantini [h], Iolanda Francolini [h], Valentina Cuzzucoli Crucitti [i], Veeren M. Chauhan [b], Robert J. Cavanagh [b], and Vincenzo Taresco[*a]

[a] School of Chemistry, University Park, Nottingham, NG7 2RD, United Kingdom.

[b] School of Pharmacy, University of Nottingham, Boots Sciences Building, University Park, Nottingham, NG7 2RD, United Kingdom.

[c] Institute for Chemical Technology of Organic Materials, Johannes Kepler University Linz, Altenbergerstrasse 69 4040, Linz, Austria.

[d] Department of Chemical Engineering and Biotechnologies, Aston University, Aston Triangle, Birmingham, B4 7ET, United Kingdom .

[e] Aston Institute for Membrane Excellence, Aston University, Aston Triangle, Birmingham, B4 7ET, United Kingdom.

[f] Department of Biochemistry, School of Science and Technology, Nottingham Trent University, Nottingham, NG11 8NS, United Kingdom.

[g] Institut de Chimie et des Matériaux Paris-Est (ICMPE), CNRS, University Paris Est Créteil, UMR 7182, 2 Rue Henri Dunant, Thiais 94320, France .

[h] Dept. of Chemistry, Sapienza University of Rome, Piazzale A. Moro 5, Rome 00185, Italy.

[I] Department of Chemical and Environmental Engineering, Faculty of Engineering, University of Nottingham, University Park, Nottingham, NG7 2RD, UK.

*Corresponding author, email: vincenzo.taresco@nottingham.ac.uk

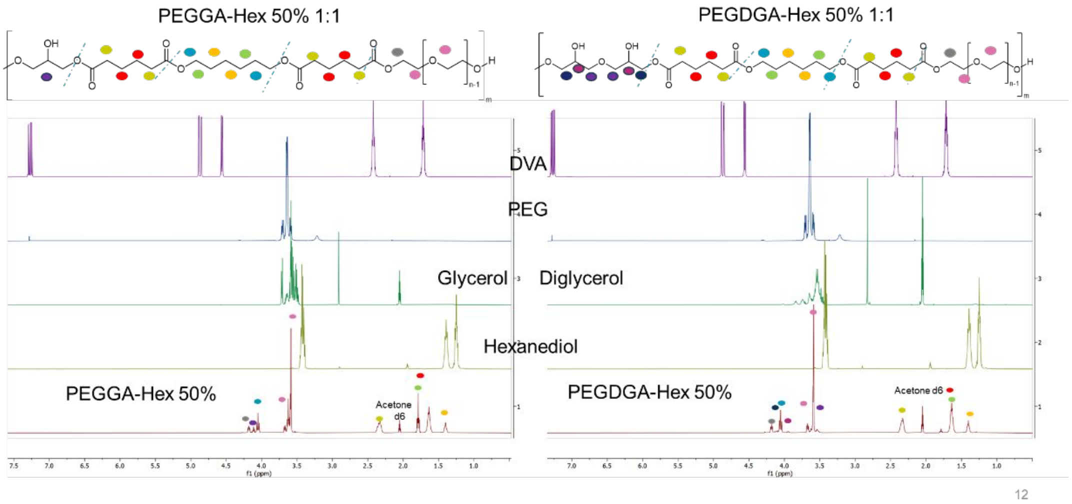

Figure S1: ^{1}H-NMR spectra of tetrapolymers spectra (polymer structure is reported with key chemical features and not in full to maximise space).

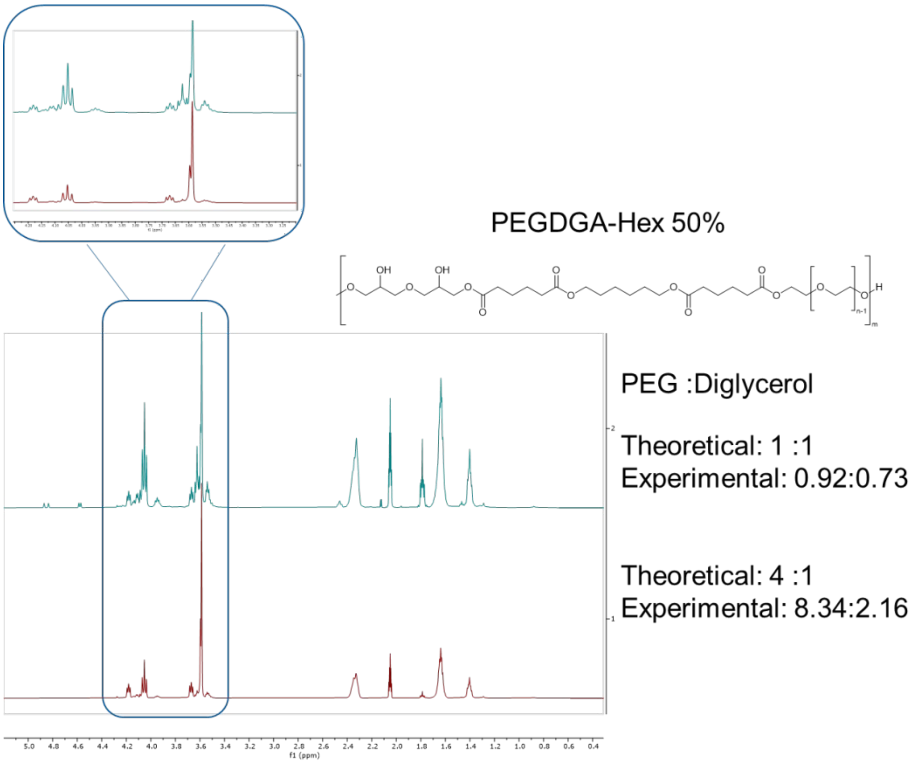

Figure S2 : ^{1}H-NMR spectra of the two different ratios of the diglycerol-based tetrapolymer PEGDGA-Hex 50%. The two ratios are 1:1 and 4:1 for PEG:Diglycerol, and the theoretical and experimental integrals of the diglycerol peaks at 3.4, 3.9, and 4.1 ppm and the PEG peaks at 3.7, 3.6, and 4.2 ppm have been compared.

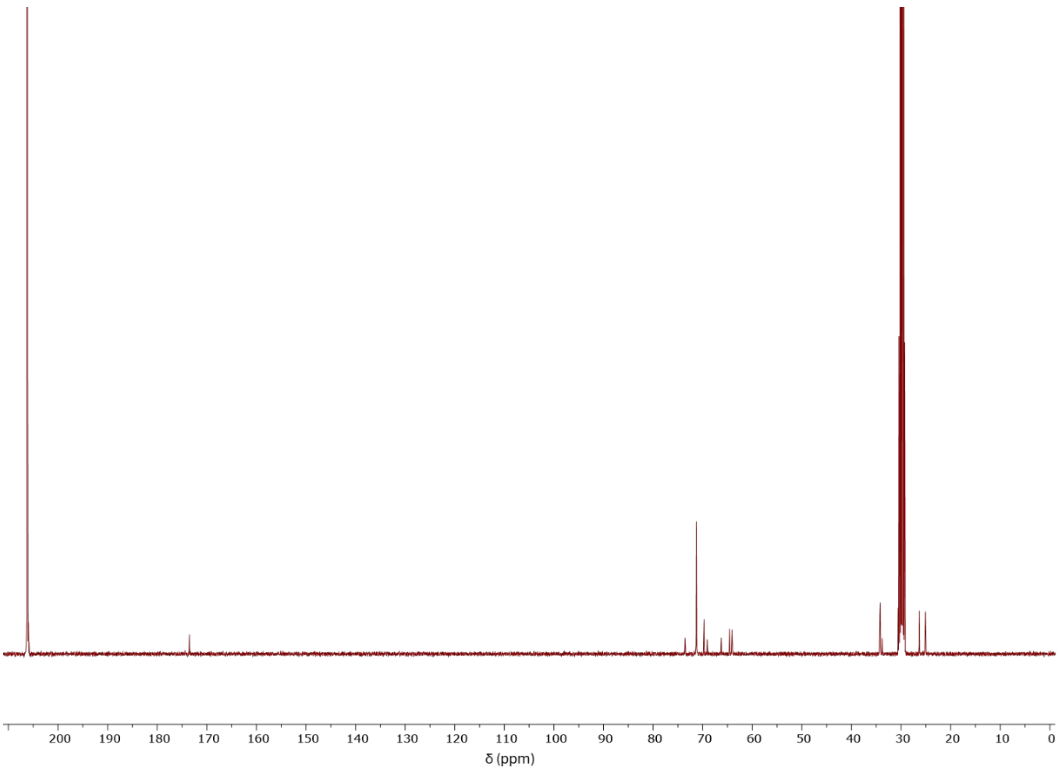

Figure S3:^{13}*C NMR spectrum of PEGDGA-Hex 50% 1:1.*

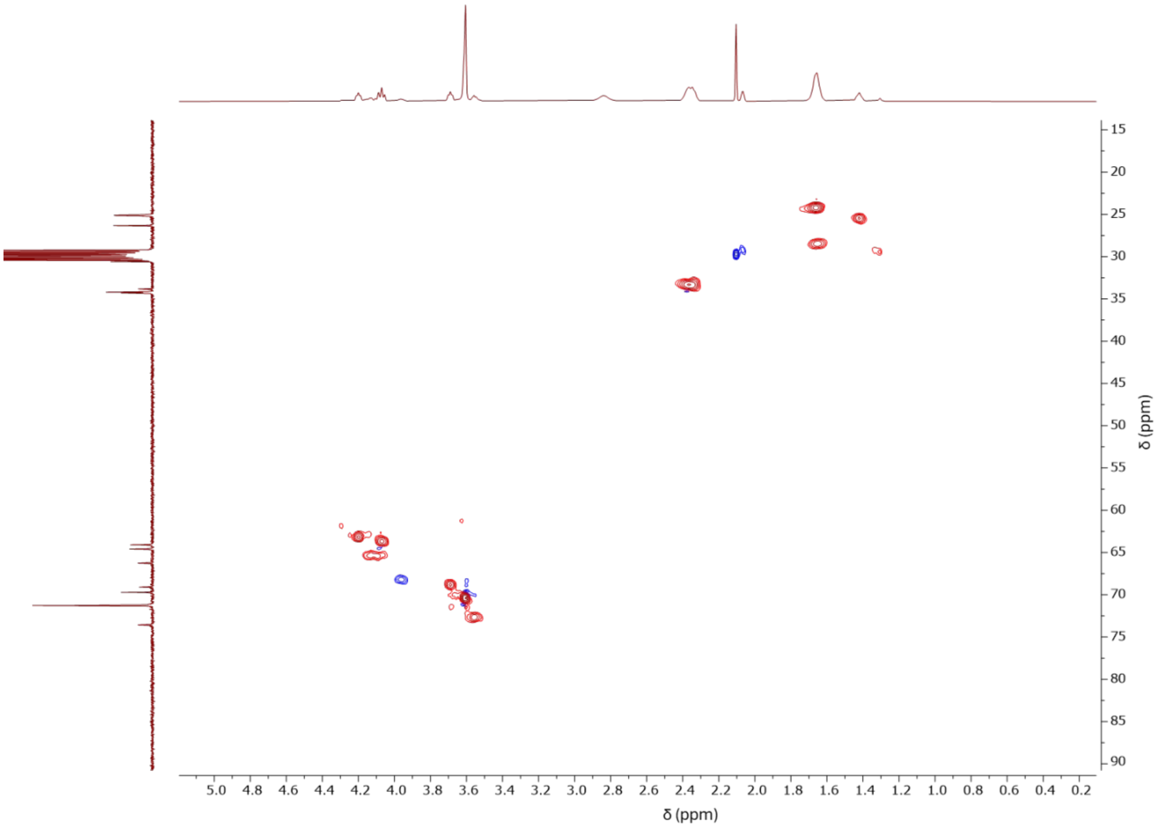

Figure S4: Multiplicity edited HSQC NMR spectrum of PEGDGA-Hex 50% 1:1.

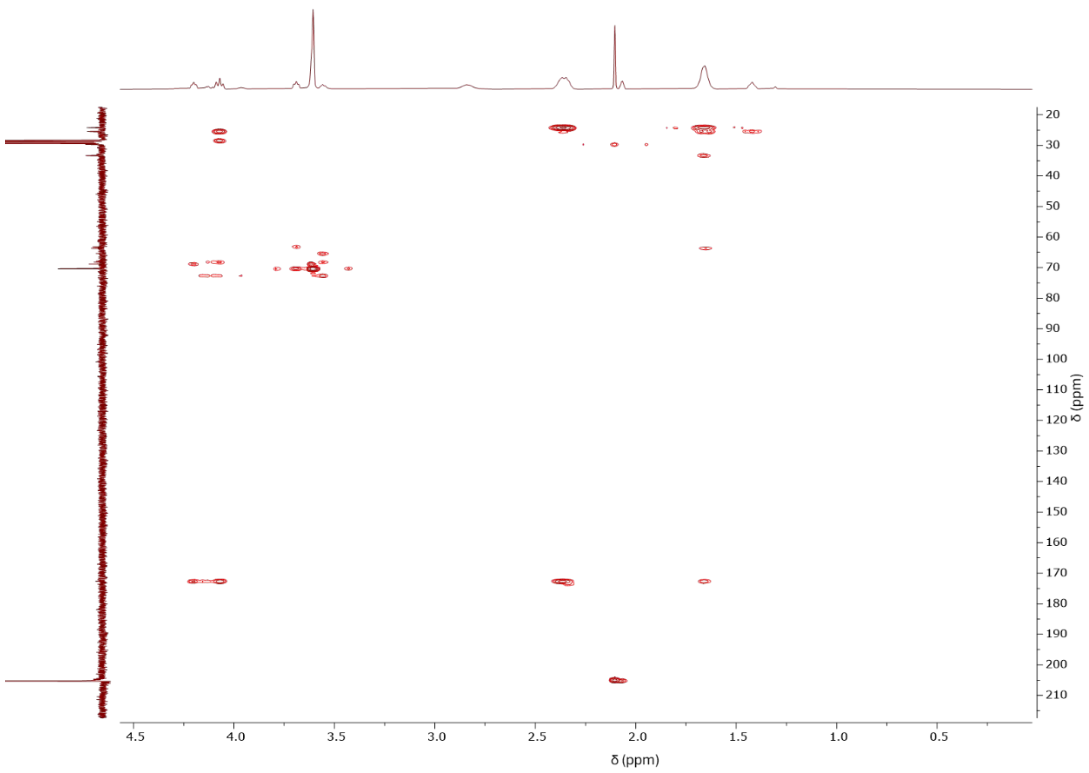

Figure S5: HMBC NMR spectrum of PEGDGA-Hex 50% 1:1.

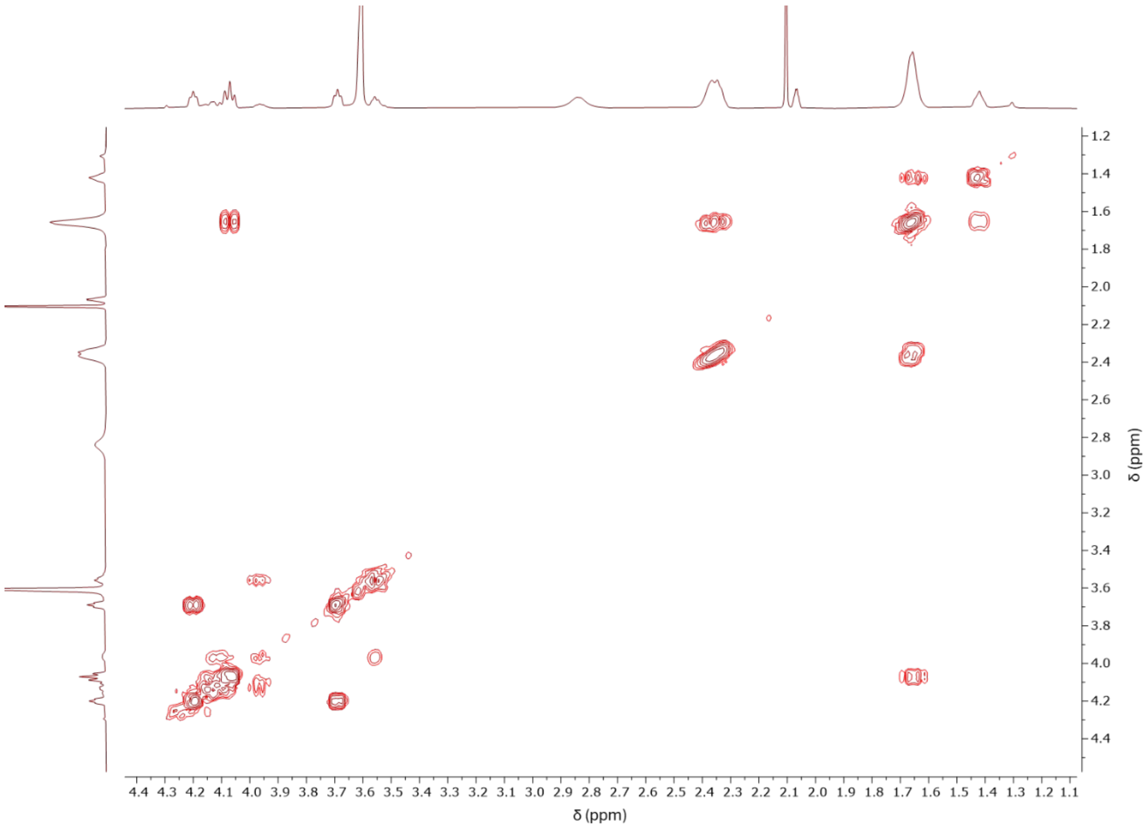

Figure S6:COSY NMR spectrum of PEGDGA-Hex 50% 1:1.

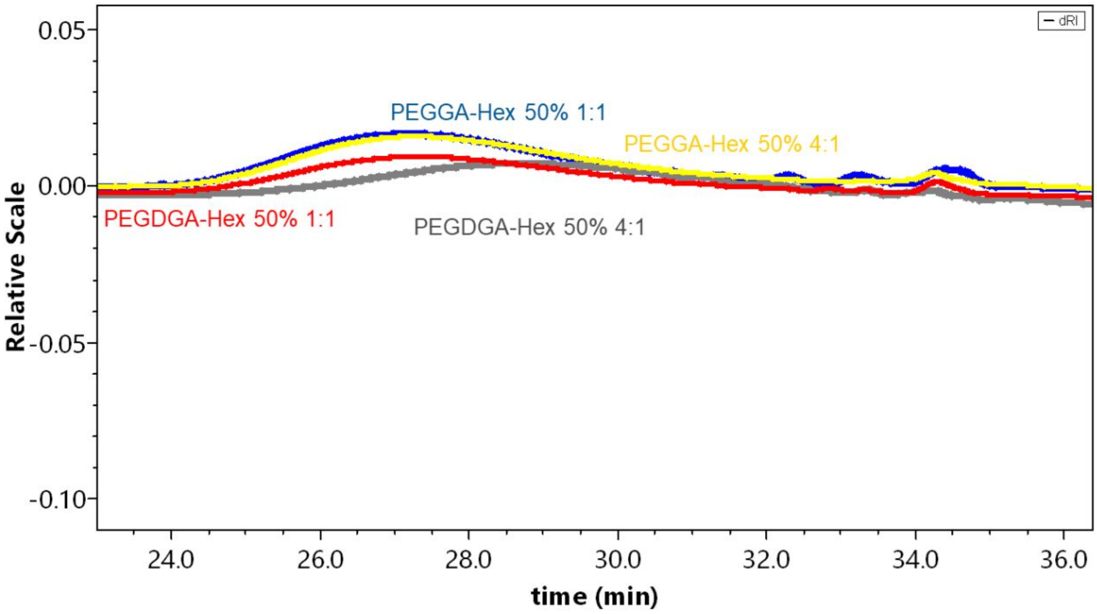

Figure S7: GPC traces of the tetrapolymers.

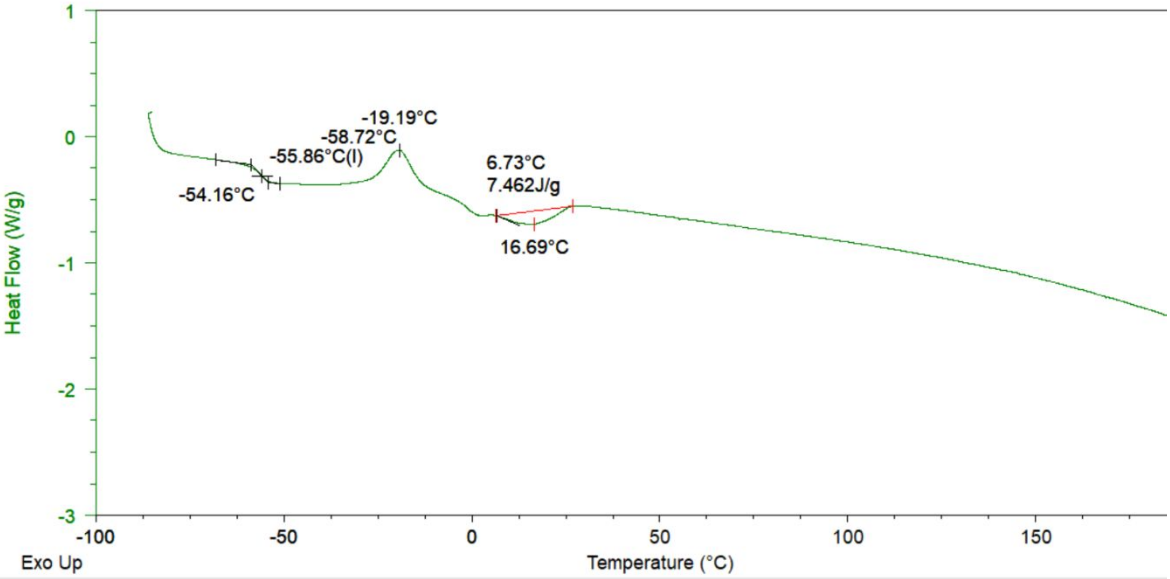

Figure S8: DSC Trace of PEGDGA-Hex 50% 1:1.

Table S 1: Z-Average, PDI , and attenuator values of the tetrapolymer NPs of unloaded and the curcumin-encapsulated polymer NPs.

Polymer	Drug	Z-Average	PDI	Attenuator
PEGGA-Hex 50% 1:1	N/A	150.5 ± 6.0	0.26 ± 0.01	9
	Curcumin	283.6 ± 3.3	0.11 ± 0.03	7
PEGGA-Hex 50% 4:1	N/A	260.0 ± 17.3	0.35 ± 0.37	7
	Curcumin	334.9 ± 13.7	0.07 ± 0.10	7
PEGDGA-Hex 50% 1:1	N/A	213.0 ± 5.2	0.08 ± 0.04	7
	Curcumin	246.0 ± 9.9	0.09 ± 0.04	6
PEGDGA-Hex 50% 4:1	N/A	272.5 ± 15.5	0.46 ± 0.49	9
	Curcumin	299.0 ± 1.9	0.09 ± 0.07	7

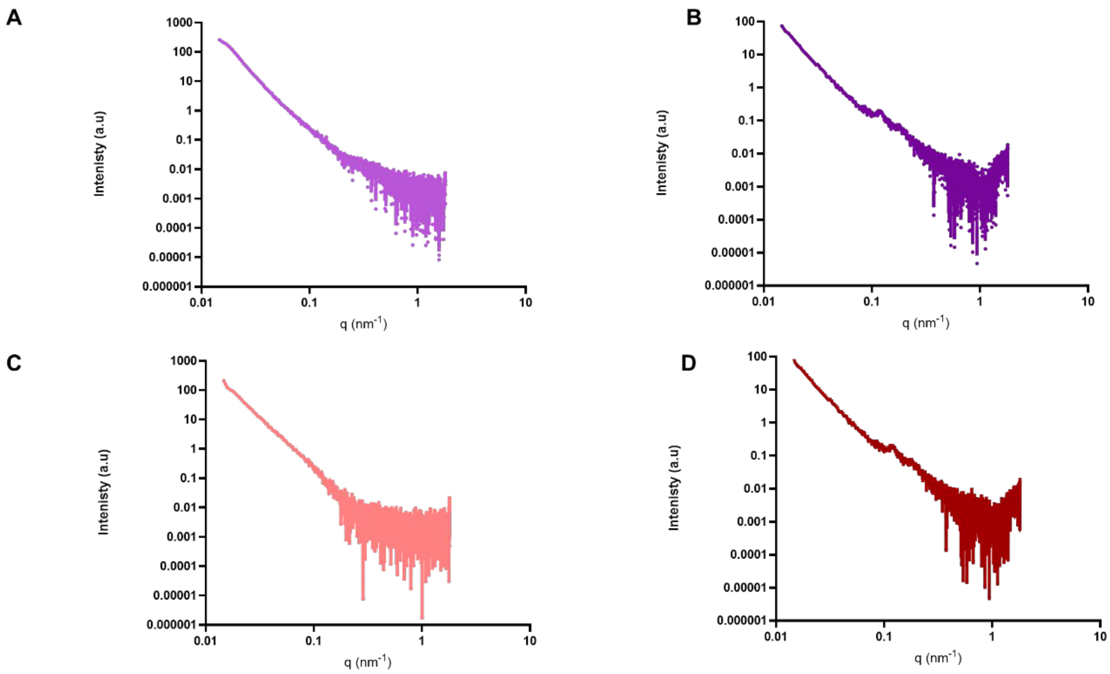

Figure S 9: SAXS analysis of the polymeric systems ***A****. PEGA-Hex 50%,* ***B.*** *PEGA-Hex 50% +Curcumin,* ***C****. PEGDGA-Hex 50%,* ***D.*** *PEGDGA-Hex 50% +Curcumin, as solid dispersions. Experimental scattering profiles.*

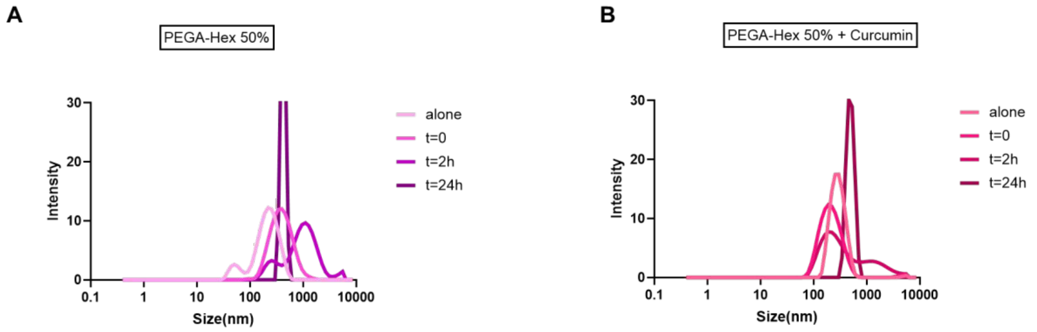

Figure S10: DLS sizes of PEGDGA-Hex 50% 1:1 during the stability studies under biologically relevant conditions in the presence of BSA for ***A.*** *PEGA-Hex 50% unloaded NPs and* ***B.*** *Curcumin-encapsulated PEGA-Hex 50% 1:1.*

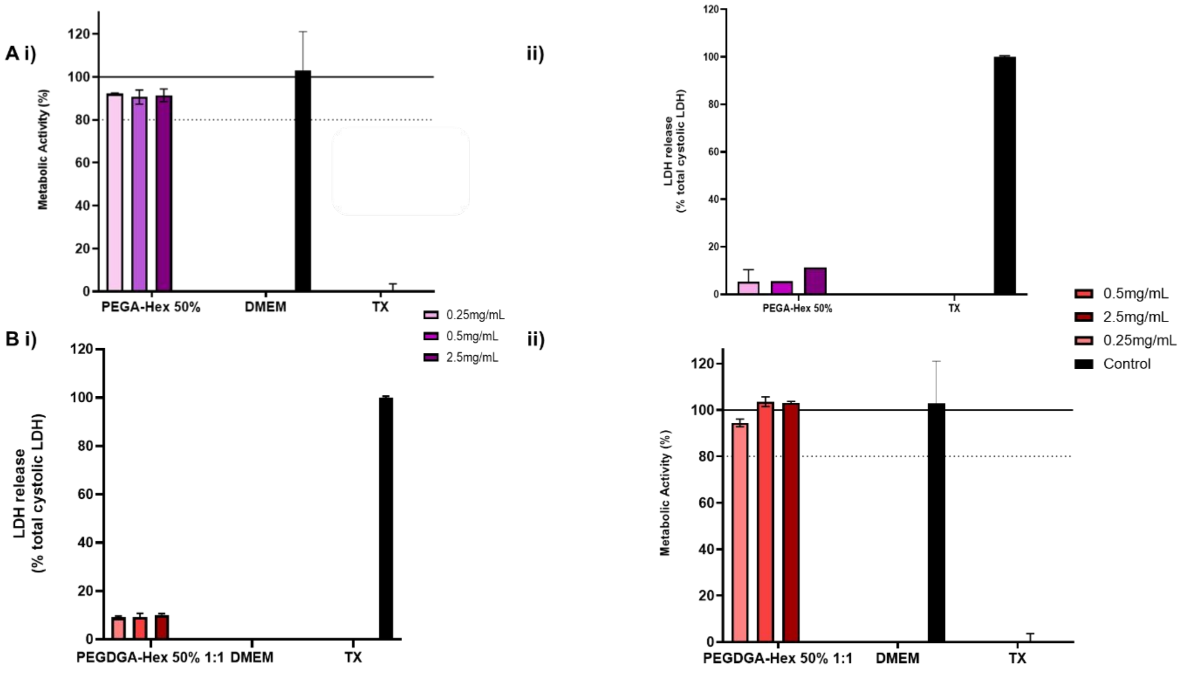

Figure S11: Cytotoxicity of polymer NPs in ***i)*** *MCF-7 human breast cancer cells and* ***ii)*** *MDA-MB-231 late-stage triple-negative breast cancer cells assessed via* ***A****. metabolic activity using PrestoBlue assay and* ***B****. LDH release assay. Cells were exposed for 24 h to varying concentrations of polymer NPs. DMEM culture media was used as vehicle control and 1 % (v/v) Triton X-100 (TX) as cell death-inducing control. Data is presented as mean ± S.D. Statistical analysis using a two-way ANOVA test determined no significant difference between the control and the polymeric samples.*

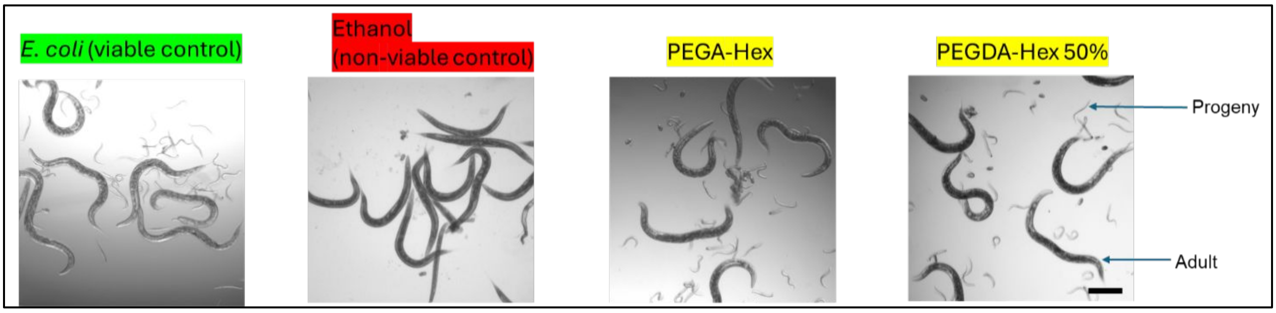

Figure S12: Representative microscope images of C. elegans 24 h after exposure to polymer nanoparticles (NPs) and control treatments. Worms exposed to E. coli and polymer NPs remained motile and produced progeny, indicating viability. In contrast, worms exposed to ethanol were non-viable, as evidenced by absence of motility, lack of response to stimuli, and no progeny production. Scale bar = 250 µm.

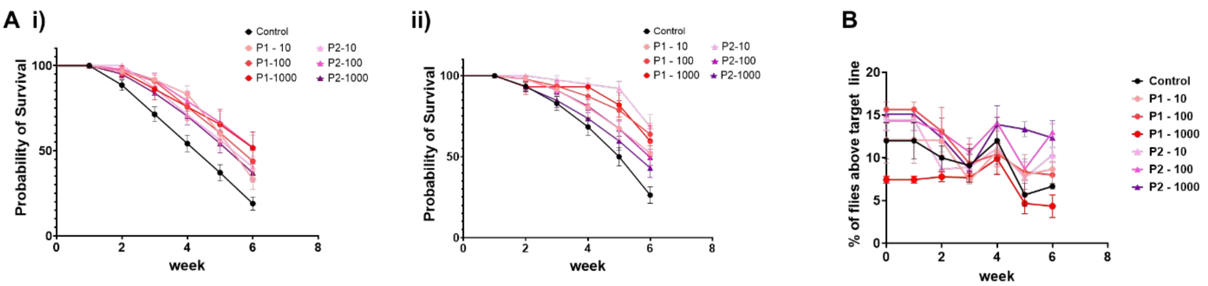

Figure 13: ***A.*** *Survival rate of F1 flies i) females and ii) males over a six-week exposure period to P1= PEGDA-Hex 50% and P2 = PEGA-Hex 50% 1:1 at different concentrations, compared with untreated controls. Survival declined progressively with age in all groups, with no significant polymer-related effects observed. Data are presented as mean ± S.D.* ***B.*** *Climbing assay (for male flies) evaluating locomotor performance after exposure. No statistically*

significant differences were detected between polymer-treated flies and controls, indicating preserved motor function.
